# Prediction of Binding Pose and Affinity of Nelfinavir, a SARS-CoV-2 Main Protease Repositioned Drug, by Combining Docking, Molecular Dynamics, and Fragment Molecular Orbital Calculations

**DOI:** 10.1021/acs.jpcb.3c05564

**Published:** 2024-03-04

**Authors:** Yuma Handa, Koji Okuwaki, Yusuke Kawashima, Ryo Hatada, Yuji Mochizuki, Yuto Komeiji, Shigenori Tanaka, Takayuki Furuishi, Etsuo Yonemochi, Teruki Honma, Kaori Fukuzawa

**Affiliations:** † Department of Physical Chemistry, School of Pharmacy and Pharmaceutical Sciences, Hoshi University, 2-4-41 Ebara, Shinagawa-ku, Tokyo 142-8501, Japan; ‡ Graduate School of Pharmaceutical Sciences, Osaka University, 1-6 Yamadaoka, Suita, Osaka 565-0871, Japan; § Department of Chemistry and Research Center for Smart Molecules, Faculty of Science, Rikkyo University, 3-34-1 Nishi-ikebukuro, Toshima-ku, Tokyo 171-8501, Japan; ∥ Institute of Industrial Science, University of Tokyo, 4-6-1 Komaba, Meguro-ku, Tokyo 153-8505, Japan; ∇ Health and Medical Research Institute, AIST, Tsukuba Central 6, Tsukuba, Ibaraki 305-8566, Japan; 6 Graduate School of System Informatics, Department of Computational Science, Kobe University, 1-1 Rokkodai, Nada-ku, Kobe 657-8501, Japan; 7 RIKEN Center for Biosystems Dynamics Research, 1-7-22 Suehiro-cho, Tsurumi-ku, Yokohama, Kanagawa 230-0045, Japan; 8 Department of Biomolecular Engineering, Graduate School of Engineering, Tohoku University, 6-6-11 Aoba, Aramaki, Aoba-ku, Sendai 980-8579, Japan

## Abstract

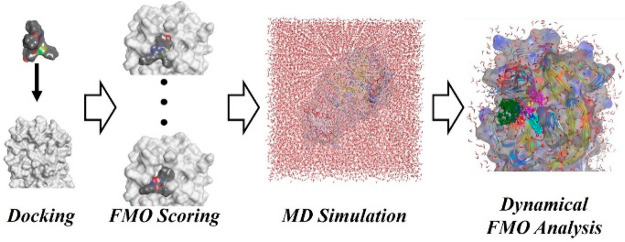

A novel *in silico* drug design procedure is described targeting the Main protease (Mpro) of the SARS-CoV-2 virus. The procedure combines molecular docking, molecular dynamics (MD), and fragment molecular orbital (FMO) calculations. The binding structure and properties of Mpro were predicted for Nelfinavir (NFV), which had been identified as a candidate compound through drug repositioning, targeting Mpro. Several poses of the Mpro and NFV complexes were generated by docking, from which four docking poses were selected by scoring with FMO energy. Then, each pose was subjected to MD simulation, 100 snapshot structures were sampled from each of the generated MD trajectories, and the structures were evaluated by FMO calculations to rank the pose based on binding energy. Several residues were found to be important in ligand recognition, including Glu47, Asp48, Glu166, Asp187, and Gln189, all of which interacted strongly with NFV. Asn142 is presumably regarded to form hydrogen bonds or CH/π interaction with NFV; however, in the present calculation, their interactions were transient. Moreover, the *tert*-butyl group of NFV had no interaction with Mpro. Identifying such strong and weak interactions provides candidates for maintaining and substituting ligand functional groups and important suggestions for drug discovery using drug repositioning. Besides the interaction between NFV and the amino acid residues of Mpro, the desolvation effect of the binding pocket also affected the ranking order. A similar procedure of drug design was applied to Lopinavir, and the calculated interaction energy and experimental inhibitory activity value trends were consistent. Our approach provides a new guideline for structure-based drug design starting from a candidate compound whose complex crystal structure has not been obtained.

## 1 Introduction

COVID-19 has been prevalent worldwide since 2019, infecting >767 million people and killing> 6.94 million as of June 2023.^1^ The causative virus, severe acute respiratory syndrome coronavirus type 2 (SARS-CoV-2), is an enveloped, positive-sense, single-stranded RNA virus belonging to the genus Betacoronavirus. The viral proteins include four structural proteins that form virus particles and 16 nonstructural proteins produced in host cells.^2^ During the four-year pandemic, scientists researched potential therapeutic agents, and their efforts resulted in the development of several effective drugs. The Food and Drug Administration (FDA) approved or authorized Paxlovid (Ritonavir-Boosted-Nirmatrelvir),^3^ remdesivir,^4^ molnupiravir,^5^ tocilizumab,^6^ baricitinib,^7^ and others.^8^ Furthermore, Ensitrelvir, an oral treatment targeting Mpro, developed in Japan, was launched in 2022. Ensitrelvir is developed by structure-based drug design using virtual screening based on docking calculations.^9^ In a new pandemic, drug repositioning is extremely important^10,11^ because in emergency the first choice is to use existing drugs whose safety and pharmacokinetics have already been confirmed in humans. The second choice is to find an existing drug or a chemical substance once regarded as a drug candidate and use it as a lead compound. Among the approved drugs, remdesivir was proposed by drug repositioning targeting SARS-CoV-2 RNA-dependent RNA polymerase, was observed to be effective,^12^ and was approved for special cases. Baricitinib was discovered by drug repositioning from a drug for rheumatoid arthritis.^13^ Moreover, the main protease (Mpro) attracted attention as a drug repositioning target by several groups.^14−17^ For example, lopinavir (LPV)/ritonavir combination drug^18,19^ and the HIV-1 Protease inhibitor Nelfinavir (NFV) have been reported to have Mpro inhibitory activity.^20^ Repositioning of NFV has not yet been successful in the development of COVID-19 therapeutics in part because of the unavailability of the NFV-Mpro complex. To obtain a plausible structure, docking calculations and Molecular Mechanics based Molecular Dynamics (MM-MD) simulations (Hereafter, referred to simply as “MD”) using the known structure as a template have been performed by several research groups, and residues such as Glu166, Gly143, and His41 have been reported to be involved in NFV binding.^21^

Thus, with recent advances in computational chemistry, the *in silico* approach is often used in the early stages of drug development. Molecular docking and MD simulations are useful methods for rational drug design, for they enable us to know molecular shapes, behaviors, prediction of binding poses and their interaction mechanisms. The docking calculation for a static structure is simpler than the MD simulations but cannot incorporate structural flexibility. In this regard, MD simulation is advantageous because it can evaluate the dynamic nature of molecules. However, since both docking and MD are based on the empirical force field, they are not quantitatively accurate enough. In contrast, quantum chemical calculations can determine the electronic state of a molecule nonempirically based on the first principles. Specifically, the fragment molecular orbital (FMO) method^22,23^ can give accurate intramolecular and intermolecular interaction energies^24,25^ based on high-speed and high-precision quantum chemical calculations for the entire protein. The FMO method has been used in drug discovery, such as protein–ligand-binding prediction^26,27^ and their interaction energy analysis.^23,28^ Comprehensive FMO calculations were performed for COVID-19-related proteins, and the resultant data have been published in the FMO database (FMODB).^29−31^

The “MD+FMO” calculation method (also called MM-MD/FMO protocol^23^), which we used in this study, combines MD and FMO calculations as follows. MD simulations were performed to sample multiple molecular structures, and FMO calculations were performed for the sampled structures. Subsequently, quantitative, and dynamic analyses were performed by averaging the obtained interaction energies. Several examples of this approach have been demonstrated to date. We performed MD+FMO calculations on the Mpro-N3 complex, the first published cocrystal structure of Mpro and its inhibitor in SARS-CoV-2.^32^ Statistical interaction analyses were performed, where 100 structures were sampled from MD simulations, and FMO calculations were performed on these structures. Thus, MD+FMO calculations for protein–ligand complexes enabled analysis of interactions between ligands and their surrounding residues while considering thermal fluctuations.^33^ In another analysis of Mpro and N3 complex,^34^ the number of structure samples was increased to 1000 and principal component analysis and singular value decomposition were performed to find the change of the relative importance of each residue through structural fluctuations. Additionally, when a similar method was used to predict protein–ligand-binding affinity for cyclin-dependent kinase-2 and seven of its ligands, the energy obtained using MD+FMO calculation showed a better correlation with the experimentally measured *ΔG* than the energy for molecular mechanics-optimized X-ray crystal structures.^35^ Therefore, MD+FMO has enabled highly accurate predictions of binding to dynamical structures.

The effect of water is also important in biomolecular simulations, but most static FMO calculations have dealt with only crystal water molecules. Continuum solvent models have been implemented in FMO calculations for protein–ligand-binding prediction. For instance, Molecular Mechanics Poisson–Boltzmann Surface Area (MM-PBSA) was applied to serine/threonine kinase Pim1 and its inhibitor,^25^ and the FMO-PB method was applied to estrogen receptor and ligand.^36^ As for the explicit solvent, MD calculation can incorporate an explicit solvent easily thanks to the solvent coordinate information obtained from its trajectory. Previous FMO studies have examined the effect of explicit hydration on the interaction energy and charge distribution in a protein,^37^ DNA,^38^ and DNA/protein complex^39,40^ evaluated the effect of hydration layer thickness on the interaction energy and performed the pair interaction energy decomposition analysis (PIEDA)^24,41^ of the interactions within the solutes and between the solute and solvent. These studies are limited to discussions of the solvent effect using only the last structure of MD trajectories, however. In the Mpro-N3^33^ and CDK2^35^ studies presented above, numerous structures were sampled from the MD trajectories, but the interactions between water and solutes were not reported in detail. Conventional quantum mechanical (QM) calculations for explicit water have been used to calculate excitation energies of small molecules^42^ and physical quantities such as proton chemical shifts in protein using QM/MM calculations,^43^ but there have been no discussions of protein–ligand-binding interactions or desolvation effects considering explicit water.

In the present study, we investigate the use of the MD+FMO method as an *in silico* drug repositioning procedure in explicitly hydrated condition. Using the Mpro and NFV complex as an example, we discuss the importance of each functional group of the ligand in binding with the protein in the light of fluctuations in molecular motion and interaction. Note that no experimental structure is available for the Mpro and NFV complex but that we effectively apply the docking simulation to model the complex. Similar calculations are also performed for LPV for comparison with experimental inhibitory activity values (IC_50_) to demonstrate the validity of our method. The present study establishes the MD+FMO method as a general procedure that provides new ideas for drug design.

## 2 Methods

**Figure 1:**
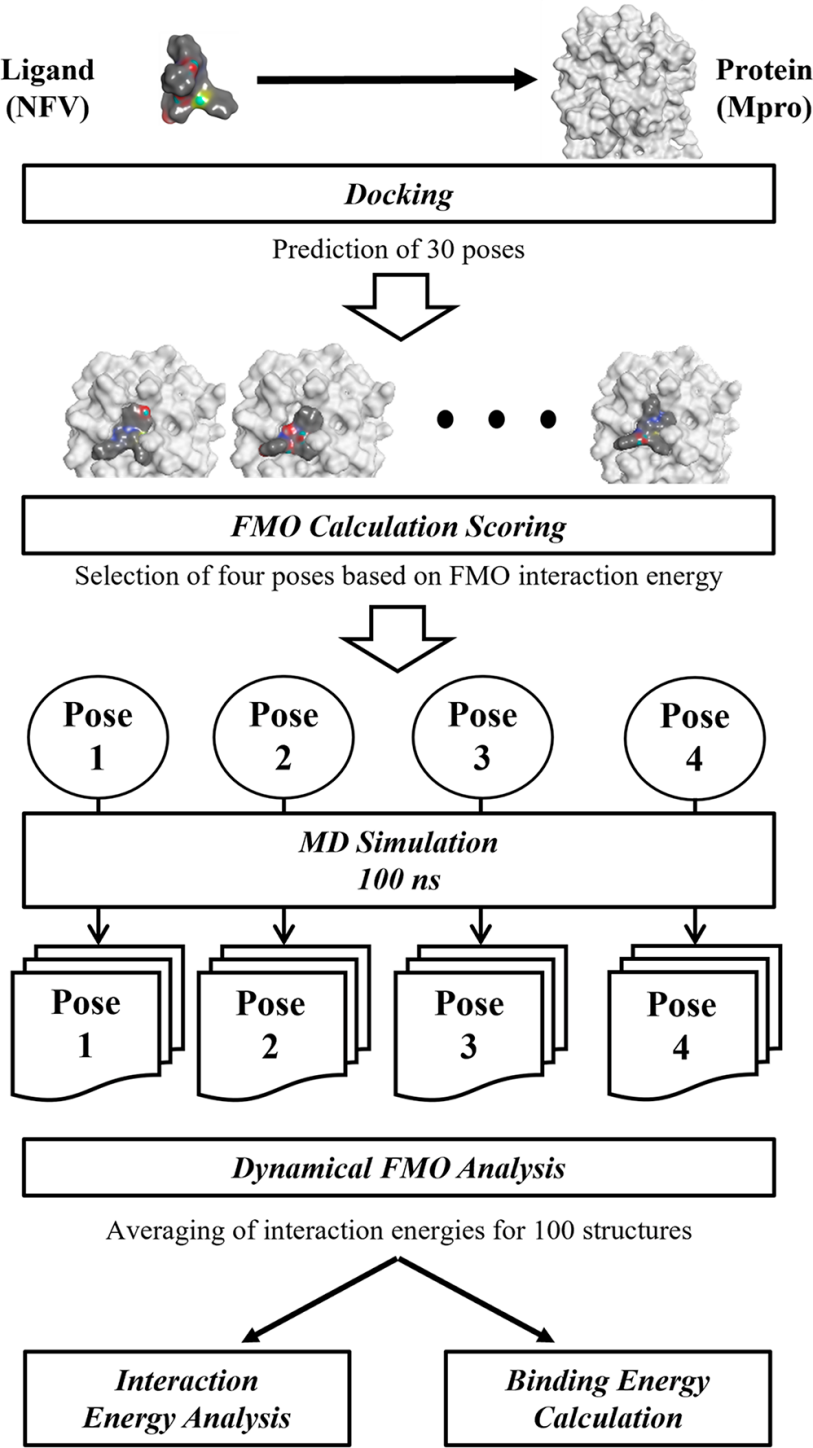
Workflow from docking calculations to “MD+FMO” calculations.

The calculation flow is shown in Figure 1. First, since the crystal structure of the NFV bound to Mpro complex has not been resolved, we predicted 30 poses by docking calculation using the cocrystal structure of similar compounds as a template. Next, we selected the dominant four poses using FMO energy scoring based on the FMO interaction energy and its binding mode. Afterward, MD simulation was performed for 100 ns each for the four poses selected. Finally, 100 structures each were sampled from the obtained trajectories, and FMO calculations were performed for 400 structures. Furthermore, interaction energy analysis was performed for each pose, and amino acid residues important for NFV recognition of Mpro were identified. Considering the deformation energy of the ligand binding to the protein and the desolvation energy in the explicit solvent, we calculated the ligand-binding energy and ranked the four poses of the complex structure from the energy score.

### 2.1 Mpro-NFV Docking Structure

**Figure 2:**
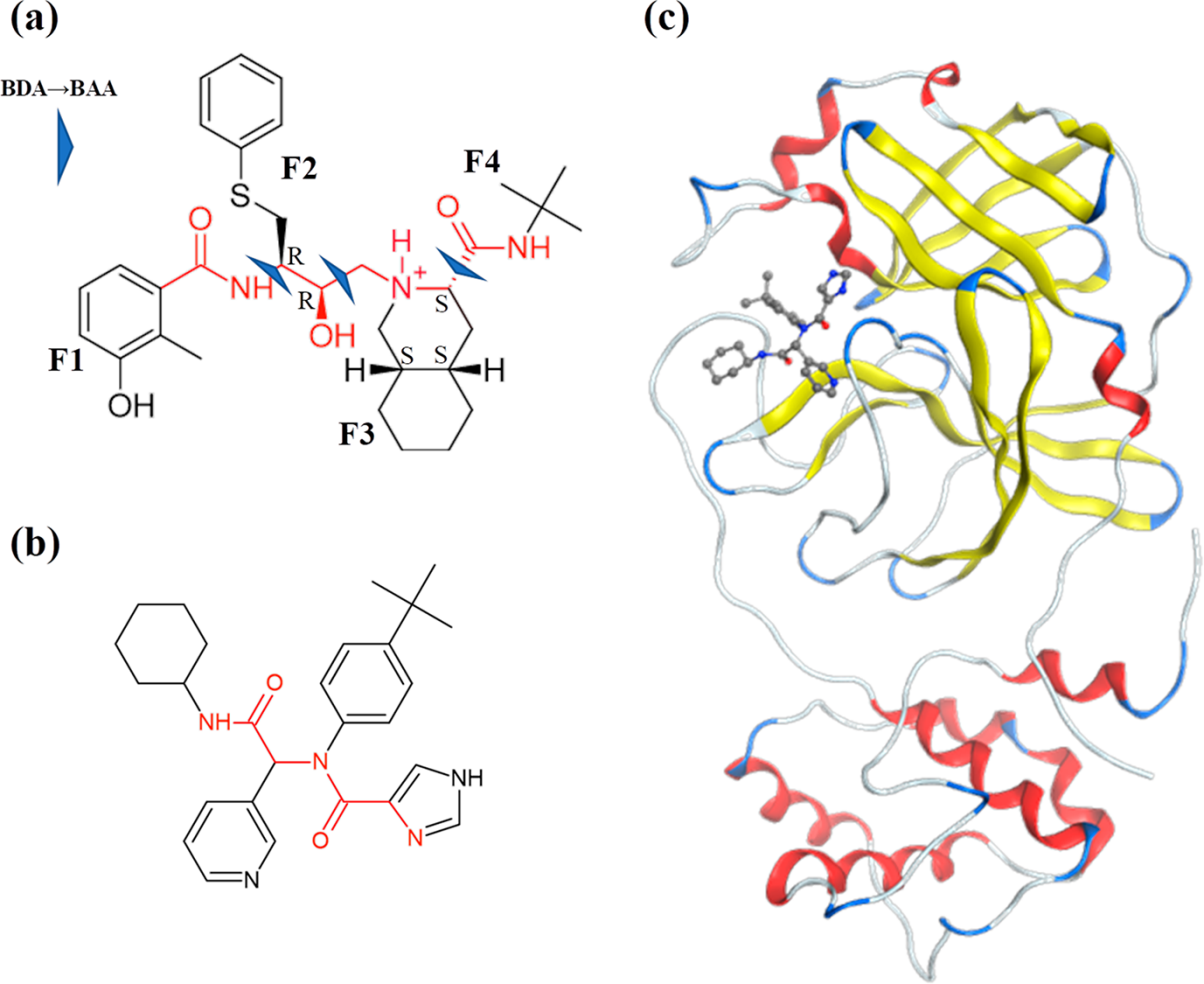
Structural formula and template structure of Nelfinavir (NFV). (a) Structural formula of NFV and fragmentation method, where F1 = fragment 1, F2 = fragment 2, F3 = fragment 3, and F4 = fragment 4. (b) NFV-like ligand. (c) Complex structure of Mpro and NFV-like ligand (PDBID: 6W63).

The structure of NFV was obtained from the Protein Data Bank (PDB) (PDBID: 3EL5).^44^ The structural formula of the NFV is shown in Figure 2a. The part indicated by the red line in the figure is a peptide-like backbone that mimics a peptide cleaved by a protease. Moreover, the cocrystal structure of the Mpro with a ligand as similar as possible to NFV was selected from PDB (PDBID: 6W63, Figure 2b,c). Since the structural formula shown in Figure 2b has a peptide-like backbone similar to NFV, some aromatic rings, a hydrophobic saturated ring, and *tert*-butyl similar to NFV, and molecular weights were also similar; hence, this complex structure was used as a template for docking calculations. Next, hydrogen atoms not determined by X-ray crystallography were added using Protonate 3D function in the Molecular Operating Environment (MOE),^45^ considering the protonation state at pH = 7.0. The protonation states of His around the ligand-binding pocket were Hie41, Hie163, Hie164, and Hie172. In ref (46), His41 was reported as Hid, suggesting that the protonation state of His can be ligand dependent. Mpro is active in its dimeric form; however, for the sake of simplicity, a monomeric protein was used in the docking calculations. We have confirmed that most residues in the ligand-binding pocket in our MD calculations of the monomeric protein retain a similar shape as in the crystal structure of the dimeric form. All crystal water molecules were eliminated. After that, the atomic coordinates were optimized. Subsequently, general docking was performed using the MOE for Mpro and NFV, and 30 poses of the complex structure were predicted. Here, the stereoisomer of NFV used in the docking calculation is the same as that in the crystal structure of the HIV-1 complex (PDBID: 3EL5), and all docking calculations were performed, including conformational searches. In addition, an induced fit was performed instead of rigid body docking. The output final score was used for comparison as the docking score. Structural refinement after docking calculation was performed with the Tether (σ) = 1.0 constraint under induced fit conditions: Tether is the standard deviation in the σ radial direction and assigns a harmonic potential to the specified atom using a force constant of (3/2)*kT*/σ^2^, where *k* and *T* are Boltzmann constant and temperature, respectively. All modeling in MOE used the AMBER10:EHT force field, which is an all-atom force field combining 2D Extended Hueckel Theory^47^ and Amber10.^48^ This force field is compatible with the RESP and AM1-BCC^49^ charges and, according to MOE, is more effective for proteins and nucleic acids than the Amber14:EHT force field.^45^ Docking structures for LPV and Mpro were also created using procedure similar to that for NFV; the Mpro and LPV structures were obtained from PDB (PDBID: 6W63 and 6DJ1, respectively).^50^

### 2.2 FMO Energy Scoring

In the FMO method, the protein complex is divided into fragments, such as amino acid residue units, and the energies of fragment monomers and fragment dimers are determined in the environmental electrostatic potential from surrounding fragments. By integrating these energies, the total energy (*E*_*total*_) and inter-fragment interaction energy (IFIE)^51−53^ can be obtained (eq 1).
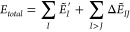
1where *Ẽ*_*I*_^′^ is the energy of the monomer, excluding the contribution of the environmental electrostatic potential, and *ΔẼ*_*IJ*_ is IFIE. Using PIEDA, IFIE can be further decomposed into the following four components (eq 2): electrostatic term (ES), exchange–repulsion term (EX), charge transfer with higher-order mixing terms (CT+mix), and dispersion term (DI).
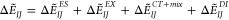
2The chemical bond can be interpreted by considering the contributions of each term. For example, hydrogen bonds are detected as stabilization energies using the ES and CT+mix terms, whereas dispersion interactions such as CH/π^54^ and π–π are detected using the DI term. In this study, Mpro was divided into each amino acid residue and NFV was divided into four fragments (F1, F2, F3, and F4) (Figure 2a). The ABINIT-MP program^51,55^ was used for FMO calculations; electron correlation effects are incorporated by the second-order Møller–Plesset perturbation (MP2) theory, which was efficiently implemented in ABINIT-MP.^56−58^ 6-31G*, standardly used in FMO calculations, was used for the basis function.^23,51^

The procedure for FMO scoring is as follows. First, for each of the 30 poses of the docking structure obtained in Section 2.1, structural optimization was performed using the AMBER10:EHT force field. Hydrogen atoms were not constrained, while amino acid side chains within 4.5 Å around the ligand were constrained by Tether = 1.0. Additionally, the coordinates of other heavy atoms (non-hydrogen atoms) were fixed. Next, the FMO calculation was performed for the optimized structure of 30 poses obtained. Lastly, scoring was performed using the sum of the IFIE of each amino acid residue of the ligand and Mpro (Total IFIE), and the top two poses showing stable values and two poses with different binding modes were selected. For FMO energy scoring, higher negative values indicate stronger and more stable interactions. Similarly, for LPV, six poses were selected from 30 poses with stable IFIE values and no overlap in binding mode (Figure S1).

### 2.3 MD Simulation

Each candidate pose obtained in Section 2.2 was subjected to MD simulation as follows. Each pose containing the protein and ligand was immersed in a periodic box of water neutralized with Na^+^ ions. The constructed molecular system was heated from 0 to 310 K for 50 ps and further simulated at 310 K for 50 ps under an *NVT* condition. The system was then simulated for an additional 101 ns under *NPT* conditions at 1013 hPa to adjust the solvent density to match the biological environment. Snapshots from the last 100 ns of the trajectory were later subjected to FMO calculations. For comparison, MD simulations were additionally performed for the protein apo structure without a ligand and for the ligand only, under conditions similar to those for the protein–ligand complex.

MD simulations were performed with the AMBER16 program^59^ using molecular topology files constructed with MOE. Temperature and pressure were kept constant with the Langevin thermostat and Berendsen barostat (time constant = 1 ps),^60^ respectively. The time integration step was 1 fs. Covalent bonds in protein and ligand were allowed to evolve freely without constraint. The TIP3P water was used as the solvent, and the protein, ligand, and ions were modeled using the AMBER10:EHT force field,^47,48^ which is the default force field in the MOE package. This choice was made to maintain consistency with our previous studies.^33^ The electrostatic interaction was calculated using the particle mesh Ewald method^61^ with a cutoff distance of 12 Å for the real-space summation.

### 2.4 Dynamical FMO Calculation

For each 100 ns trajectory of the four complexes obtained using MD simulation, 100 structures were extracted at 50 ps intervals from the latter half of 50 ns. Therefore, 400 structures were extracted, and the FMO calculation was performed. In the case of LPV, it was performed on 600 structures of six poses. Each sample structure was extracted to a droplet form with water molecules within 4 Å of Mpro; this criterion of water layer thickness was determined following refs (37−40). Before FMO calculations were performed, the geometry was optimized using the AMBER10:EHT force field for each sampling structure. As in the method shown in Section 2.2, hydrogen was not constrained. However, amino acid side chains within 4.5 Å around the ligand were constrained by Tether = 1.0, and the coordinates of other heavy atoms were fixed. Next, FMO calculation was performed at the MP2/6-31G* level using the ABINIT-MP Program. Additionally, the FMO calculation was performed under similar conditions for the protein apo structure containing no ligand. Subsequently, the average value and standard deviation of 100 structures of total IFIE and PIEDA with Mpro for NFV obtained by using these FMO calculations were calculated for each pose. Additionally, since NFV is divided into four fragments (Figure 2a), interaction analysis was performed by calculating the average value and standard deviation of 100 structures for IFIE and PIEDA for each fragment.

### 2.5 Ligand-Binding Energy Calculation

The binding energy (Δ*E*_*bind*_) is expressed as the sum of the protein–ligand intermolecular interaction energy (*ΔE*^*int*^), the deformation energy of the ligand (Δ*E*_*lig*_^*def*^), and the solvation energies (*ΔE*^*sol*^)^25,36^ (eq 3). Additionally, *ΔE*^*int*^ is the total energy of the complex (*E*_*com*_) minus the total energy of the protein alone (*E*_*pro*_) and the ligand alone (*E*_*lig*_) (eq 4). Here, the sum of the IFIEs of the ligand in the complex and each amino acid residue (total IFIE; ∑Δ*Ẽ*_*IJ*_) is used for approximation (eq 5). Additionally, here, *I* indicate the ligand fragment (NFV) and *J* indicates each amino acid fragment of the protein (Mpro). Furthermore, Δ*E*_*lig*_^*def*^ is the deformation energy of the ligand structure, indicated by the difference in total energy between the complexed (com) and isolated (sol) forms in water (eq 6). The average of 100 structures for each pose was used to calculate Δ*E*_*lig*_^*def*^. Moreover, the isolated structure was optimized with B97D^62^/6-31G* using Gaussian16, and single-point calculation was performed with MP2/6-31G* using ABINIT-MP (*E*_*lig(sol)*_).

Furthermore, *ΔE*^*sol*^ are the complex solvation energies minus the protein and ligand solvation energies (eq 7).
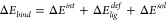
3
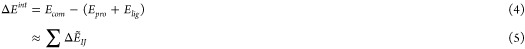
4
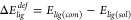
6
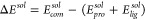
7Here, the solvation energy of the complex (*E*_*com*_^*sol*^) is half the sum of the interaction energies between ligand and water molecules (∑Δ*E*_*IK*_^*com*^) and between amino acids and water molecules (∑Δ*E*_*JK*_^*com*^) in the complex form (eq 8). Additionally, the protein solvation energy (*E*_*pro*_^*sol*^) is half of the interaction energy between amino acids and water molecules in the apo structure (eq 9). Similarly, the ligand solvation energy (*E*_*lig*_^*sol*^) is half the interaction energy between the ligand and water molecules in the isolated free structure (eq 10). *K* indicates a water molecule fragment.
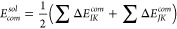
8
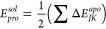
9
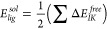
10The method for selecting the amino acid fragment (*J*) used in determining the protein solvation energy (*E*_*pro*_^*sol*^) (eq 9) is as follows: First, residues whose average distance between nearest neighbor atoms from the ligand is within 4 Å were determined in 100 complex structures sampled for each pose. This was performed for four poses, and amino acid residues targeted in one or more poses were commonly subjected to integration in all poses. Consequently, 25 amino acid residues were used (Thr25, Thr26, Leu27, His41, Ser46, Met49, Leu50, Leu141, Asn142, Gly143, Ser144, Cys145, His163, His164, Met165, Glu166, Leu167, His172, Phe181, Asp187, Arg188, Gln189, Thr190, Ala191, and Gln192) (Figure S2).

In this study, *ΔE*^*int*^ is written as Δ*E*^*int*(*static*)^ for the static structure and *ΔE*^*int*^ for the dynamic structure.

## 3 Results and Discussion

### 3.1 Scoring Using Docking and FMO Calculations

**Figure 3:**
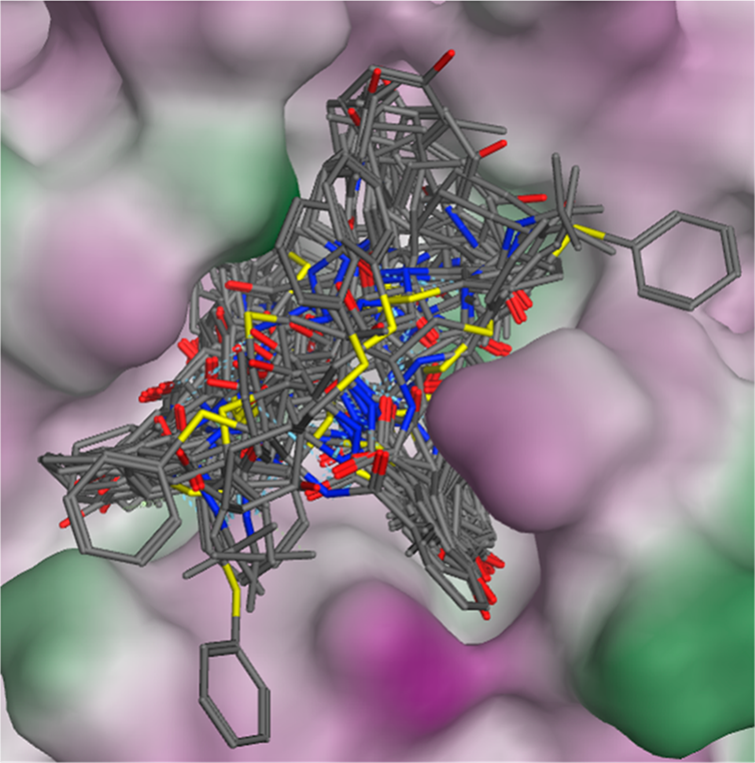
Superposition of 30 structures obtained by docking Mpro and NFV. Among the ligand-binding pockets of Mpro, purple indicates hydrophilic and green indicates lipophilic.

**Figure 4:**
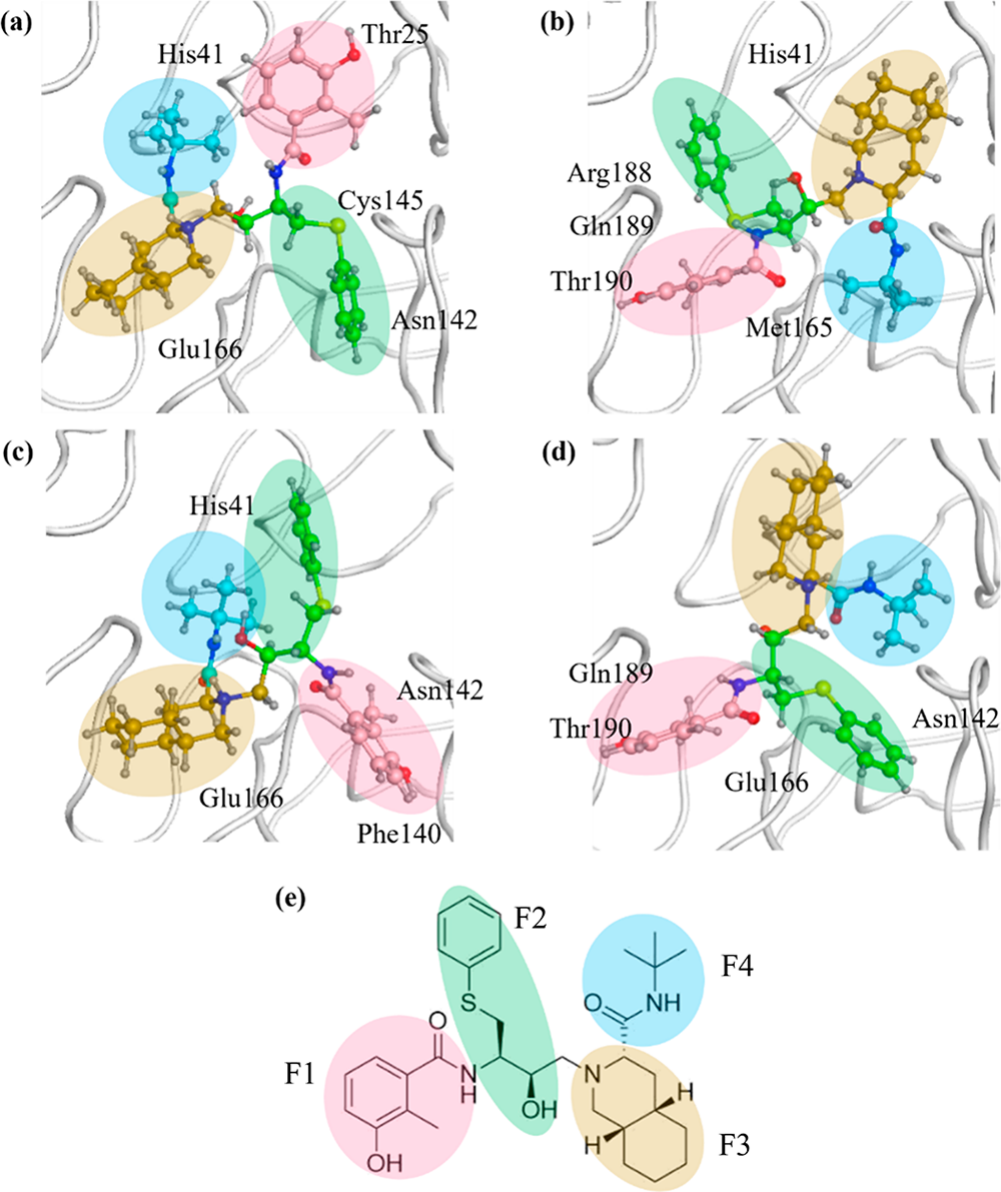
Four structures selected based on docking and FMO scoring: (a) Pose 1, (b) Pose 2, (c) Pose 3, and (d) Pose 4. (e) Coloration based on NFV fragmentation, where F1 = fragment 1, F2 = fragment 2, F3 = fragment 3, and F4 = fragment 4. The FMODB IDs of each pose are (a) 9MM52, (b) L7759, (c) 3772L, (d) M99KZ.

The 30-pose composite structure obtained by docking Mpro and NFV is shown in Figure 3. All poses were scored by FMO calculation (Table S1), and four poses with different binding modes to ligands were selected as candidate structures (Figure 4), considering the binding mode and FMO scoring results (*ΔE*^*int*(*static*)^). As presented in Table S1, the ranking by docking score is Poses 1, 2, 3, and 4 in descending order of scores, with Pose 1 having the best value. On the other hand, the ranking by FMO scoring is Poses 3, 1, 4, and 2 in descending order, with Pose 3 showing the best value. Between each pose, the IFIE value and the numbers of hydrogen and CH/π bonds based on it were different. A CH/π bond is a noncovalent dispersion interaction between a CH bond and the π-electron system. The typical distance of this intermolecular bond is 2.4–3.2 Å and is known to be about one-third the strength of a hydrogen bond.^63−65^ The Δ*E*^*int*(*static*)^ values of Poses 1–4 were −147 to −193 kcal/mol, which were more stable than that of Δ*E*^*int*(*static*)^ (Figure S3) (−145 kcal/mol) in the original complex structure with the NFV analogue (Figure 2c), used as a template for docking calculations. Here, regarding the stereoisomers of NFV, Sargolzaei et al. suggested that a stereoisomer different from PDBID 3EL5 was the optimal structure of NFV for binding to Mpro.^21^ They also noted that the presence of F1 or F3 of NFV in the anchor site of Mpro was important.^21^ The structures of Poses 1–4 obtained in this study have F1 or F3 of NFV in the anchor site of Mpro, consistent with the argument of Sargolzaei et al.

In Pose 1, we found two hydrogen bonds (SH–O bonds) at Thr25 and Cys145 with NFV and four CH/π bonds with Thr25, His41, Asn142, and Glu166. In Pose 2, we observed one hydrogen bond with Thr190 (Ala191 C=O) and three CH/π bonds with His41, Arg188, and Gln189. Notably, dispersive interactions with Met165 contributed to the stabilization. In Pose 3, two hydrogen bonds with the main chain of Phe140 and Asn142 and two CH/π bonds with His41 and Glu166 were found; further, for Asn142, in addition to a weak CH/π interaction, both π-electrons of the two amide groups in the main and side chains showed π–π interactions with the ligand phenol ring. Finally, in Pose 4, one hydrogen bond with Thr190 (Ala191 C=O) and two CH/π bonds with Glu166 and Gln189 were observed; further, for Asn142, in addition to a weak CH/π interaction, both the π-electrons showed π–π interactions with the ligand benzene ring, as in Pose 3 (Figure 4 and Figure S4). Notably, a larger number of hydrogen bonds in each pose resulted in a more stable ES term (Table 1). Similarly, a greater number of CH/π bonds and interactions between hydrophobic functional groups gave a more stable DI term.

**Table 1:** Interaction and Binding Energies of Each Docking Pose^a^

	Pose 1	Pose 2	Pose 3	Pose 4
**Docking Structure**				
docking score	–9.70	–9.63	–9.07	–9.00
ES	–150.4	–135.1	–139.5	–145.9
EX	105.1	118.1	63.2	89.9
CT + mix	–35.0	–24.4	–33.1	–33.4
DI	–103.5	–105.6	–83.1	–84.9
Δ*E*^*int*(*static*)^ (Total IFIE)	–183.7	–147.1	–192.6	–174.3
**Dynamical FMO Analysis**				
ES	–116.4 ± 23.3	–110.5 ± 20.4	–199.0 ± 23.7	–138.5 ± 18.6
EX	61.4 ± 15.2	46.7 ± 16.0	92.4 ± 18.6	79.7 ± 13.5
CT + mix	–24.8 ± 5.1	–18.2 ± 5.6	–34.6 ± 5.7	–27.1 ± 3.9
DI	–65.0 ± 10.2	–53.9 ± 9.8	–70.8 ± 6.6	–77.9 ± 6.7
Δ*E*^*int*^ (Total IFIE)	–144.8 ± 23.5	–135.9 ± 20.0	–212.0 ± 21.0	–167.2 ± 15.1
**Binding Energies**				
Δ*E*_*lig*_^*def*^	73.2 ± 10.9	70.9 ± 9.1	71.2 ± 10.9	80.2 ± 10.3
Δ*E*^*sol*^	61.5	60.2	123.5	107.6
Δ*E*^*int*^ + Δ*E*_*lig*_^*def*^	–71.6 ± 25.0	–65.0 ± 21.0	–140.7 ± 23.3	–86.9 ± 18.4
Δ*E*^*int*^ + Δ*E*^*sol*^	–83.3	–75.7	–88.5	–59.6
Δ*E*_*bind*_	–10.1	–4.8	–17.2	20.6

a Δ*E*^*int*^: sum of the protein–ligand intermolecular interaction energies, denoted as Δ*E*^*int*(*static*)^ for static structures and Δ*E*^*int*^ for dynamic structures. Δ*E*_*lig*_^*def*^: deformation energy. Δ*E*^*sol*^: solvation energy. Δ*E*_*bind*_: binding energy. Energy values are in kcal/mol.

In a previously reported FMODB COVID-19 special issue,^31^ the interaction between Mpro and various ligands was comprehensively analyzed using the X-ray cocrystal structures of 110 Mpro-Ligand complexes. Comparing the findings in that study with the present results, the hydrogen bonds with the main chain of Phe140 Asn142 seen in Pose 3 were similar.^31,66^ In contrast, the two hydrogen bonds in Pose 1 and the hydrogen bond with Thr190 seen in Poses 2 and 4 were observed in some structures but not universally.

### 3.2 MD Simulations

**Figure 5:**
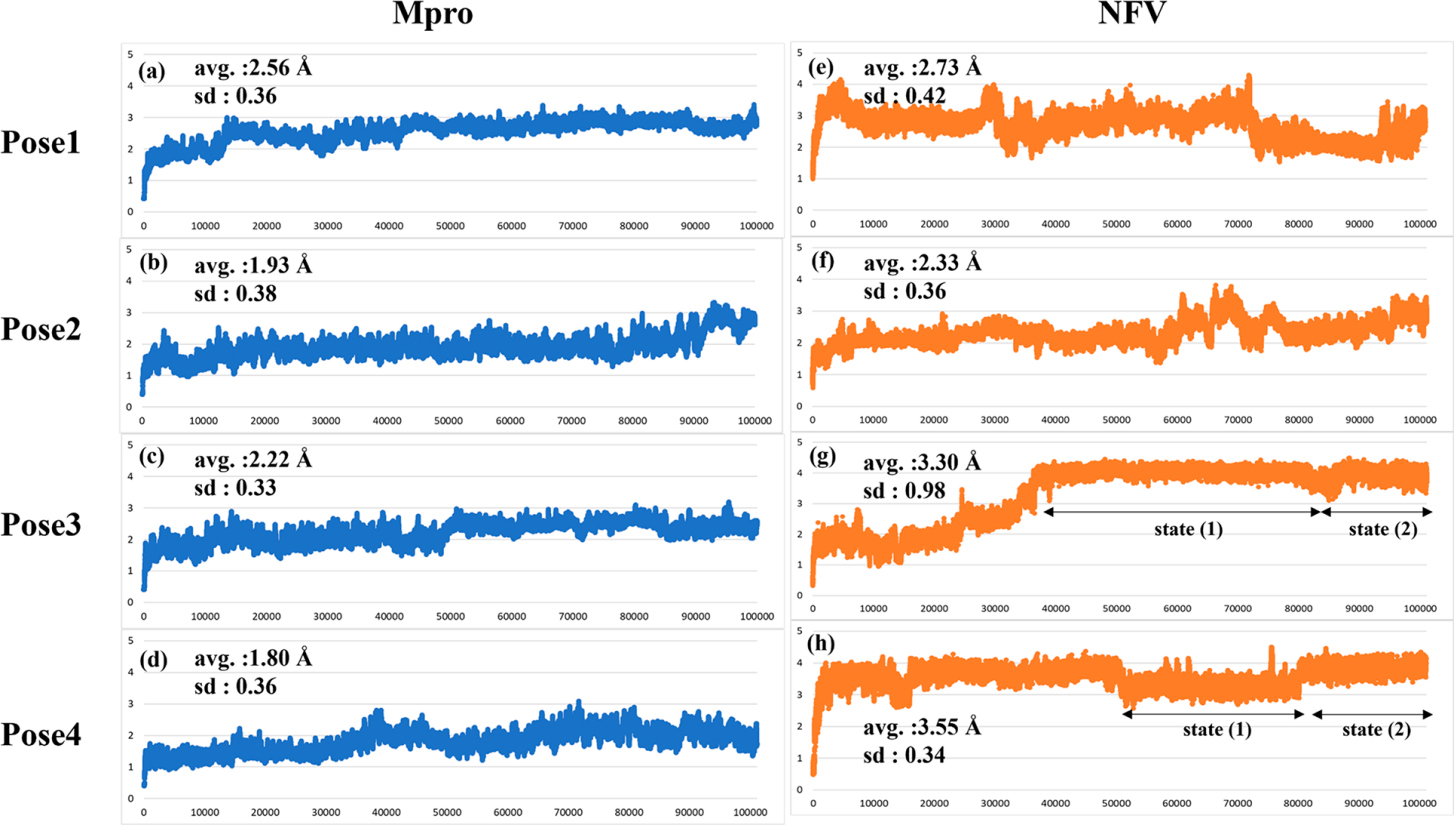
Root mean square deviation (RMSD) of protein main chain and that of ligand in the MD structure of each pose. (a)–(d) RMSD of the protein main chain in Poses 1–4. (e)–(h) RMSD of heavy atoms of NFV in Poses 1–4. The horizontal axis is time (ps), and the vertical axis is RMSD (Å).

Trajectory analysis in MD simulations from 0 to 100 ns yielded the root-mean-square deviation (RMSD) of the complex protein backbone (Figure 5a–d). The average RMSDs of protein backbone atoms (C, CA, and N) were 2.56, 1.93, 2.22, and 1.80 (Å) from Poses 1–4, sequentially and the standard deviations of RMSD were 0.36, 0.38, 0.33, and 0.36 (Å), respectively. From these results, although the structure differed from the docking structure, the standard deviation was small, indicating no significant structural change. It was also observed that most of the residues in the ligand-binding pockets, including Glu166, maintained the similar shape in all poses as in the dimeric crystal structure. Notably, the RMSD of the last 10 ns (90–100 ns) in Pose 2 was slightly >3.0. However, this is due to structural fluctuations at the C-terminus and did not affect the interaction with the NFV. The average RMSD values of NFV were 2.73, 2.33, 3.30, and 3.55 (Å) from Poses 1–4 sequentially, and the standard deviations of RMSD were 0.42, 0.36, 0.98, and 0.34 (Å), respectively. Notably, the average value indicates that the structural deformation of NFV is greater than that of Mpro in induced fit. This is particularly noticeable in Poses 3 and 4.

Moreover, when calculating the RMSD only in 50–100 ns, the average values of RMSD were 2.59, 2.52, 3.99, and 3.51 (Å), and the standard deviations of RMSD were 0.45, 0.37, 0.15, and 0.31 (Å), starting from Pose 1. Compared with the results from 0 to 100 ns, the standard deviation of RMSD does not change in Poses 1, 2, and 4; however, it is particularly small in Pose 3. The results suggest that MD in Pose 3 reached equilibrium after 50 ns, later than in the other poses. Therefore, we performed structural sampling for FMO calculation focusing on the latter half of 50 ns. The RMSD of the active site (the same residues used in Figure S2) at 51–100 ns was also measured, and each pose was compared to the apo structure of the protein. Pose 2 exhibited the same degree of pocket movement as apo (RMSD = 2.36 ± 0.33 (Å) and 3.28 ± 0.29 (Å) in Pose 2, apo, respectively), while Poses 1, 3, and 4 showed a smaller standard deviation in RMSD, about 0.13. As discussed later in Section 3.3.1, both Pose 3 and Pose 4 had two states (Figure 5g,h). These states were found from changes in the FMO interaction energies before and after MD simulations. Detailed structure analysis for the MD simulation and FMO results including multiple ligand state is described in Section 3.3.1.

### 3.3 Evaluation of Ligand Binding Using Dynamical FMO Calculation

**Figure 6:**
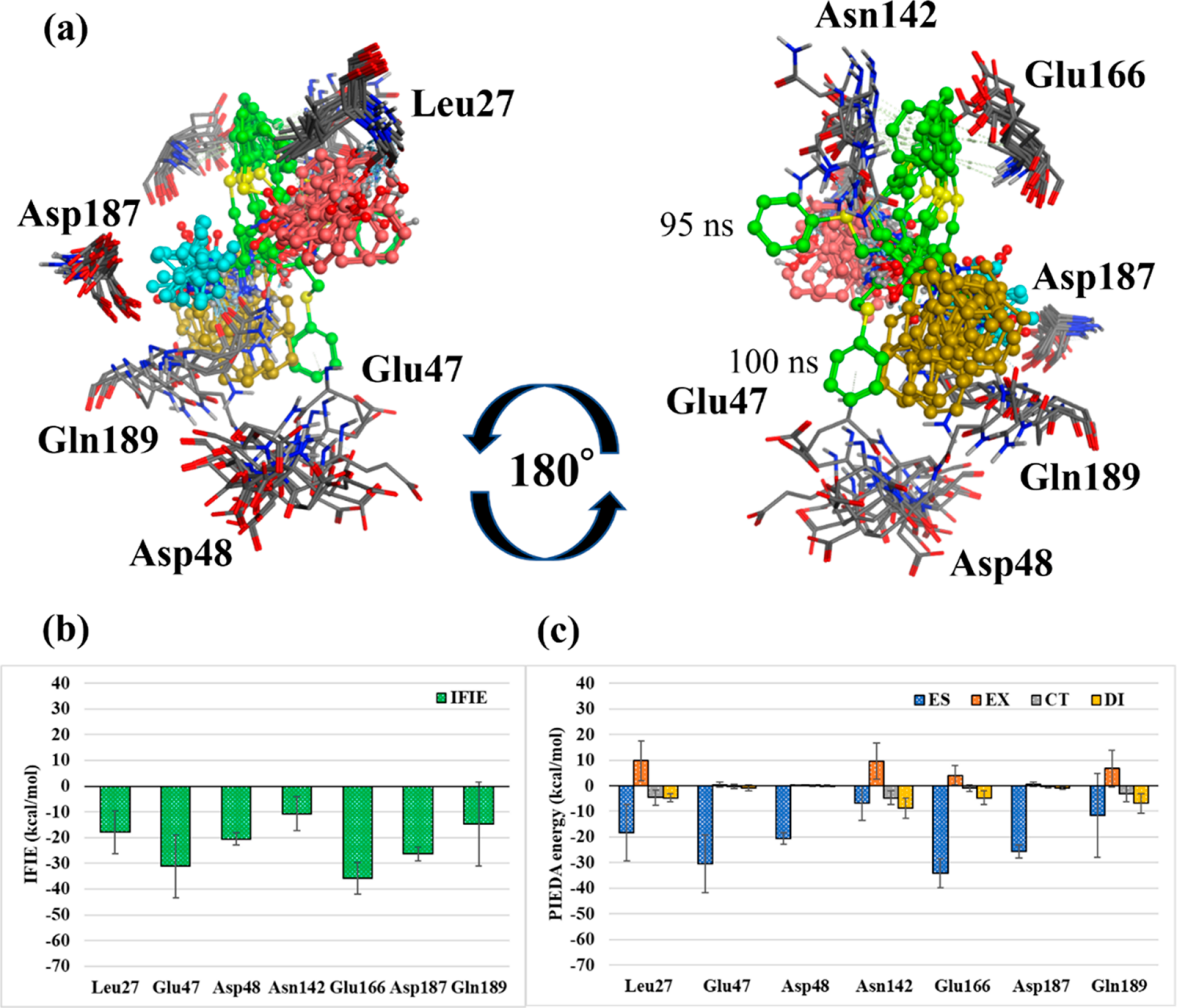
Interactions with the surrounding amino acids in Pose 1. (a) Amino acid residues of main protease interacting with Nelfinavir in Pose 1. The four ligand fragment colors: F1, pink; F2, green; F3, yellow; F4, light blue. (b),(c) Interaction energies between each amino acid residue and NFV: (b) IFIE and (c) PIEDA energies.

For each 100 sampled structures extracted from MD trajectories of four poses, the interaction and binding energy were evaluated using FMO calculations. Table 1 and Figure 6 present the average value and standard deviation of the total IFIE for each pose (Δ*E*^*int*^). The order of IFIE stability (Δ*E*^*int*^) was 3, 4, 1, and 2; hence, Pose 3 was the most stable. Although the EX term is weak in Poses 1 and 2, the stabilization energies, such as the ES and DI terms, are also weak. Moreover, Pose 3 had a stronger stabilization energy than other poses, and the ES term has a particularly dominant contribution. Furthermore, Pose 4 had a strong stabilization energy similar to that of Pose 3, and the DI term was the strongest in the four poses. However, Pose 4 was not the most stable because the ES term was weaker than that of Pose 3. The characteristics of each pose are discussed as follows.

#### 3.3.1 Fluctuations in the Mpro-NFV Bond Structure and Changes in Their Interactions

##### 3.3.1.1 Pose 1

**Figure 7:**
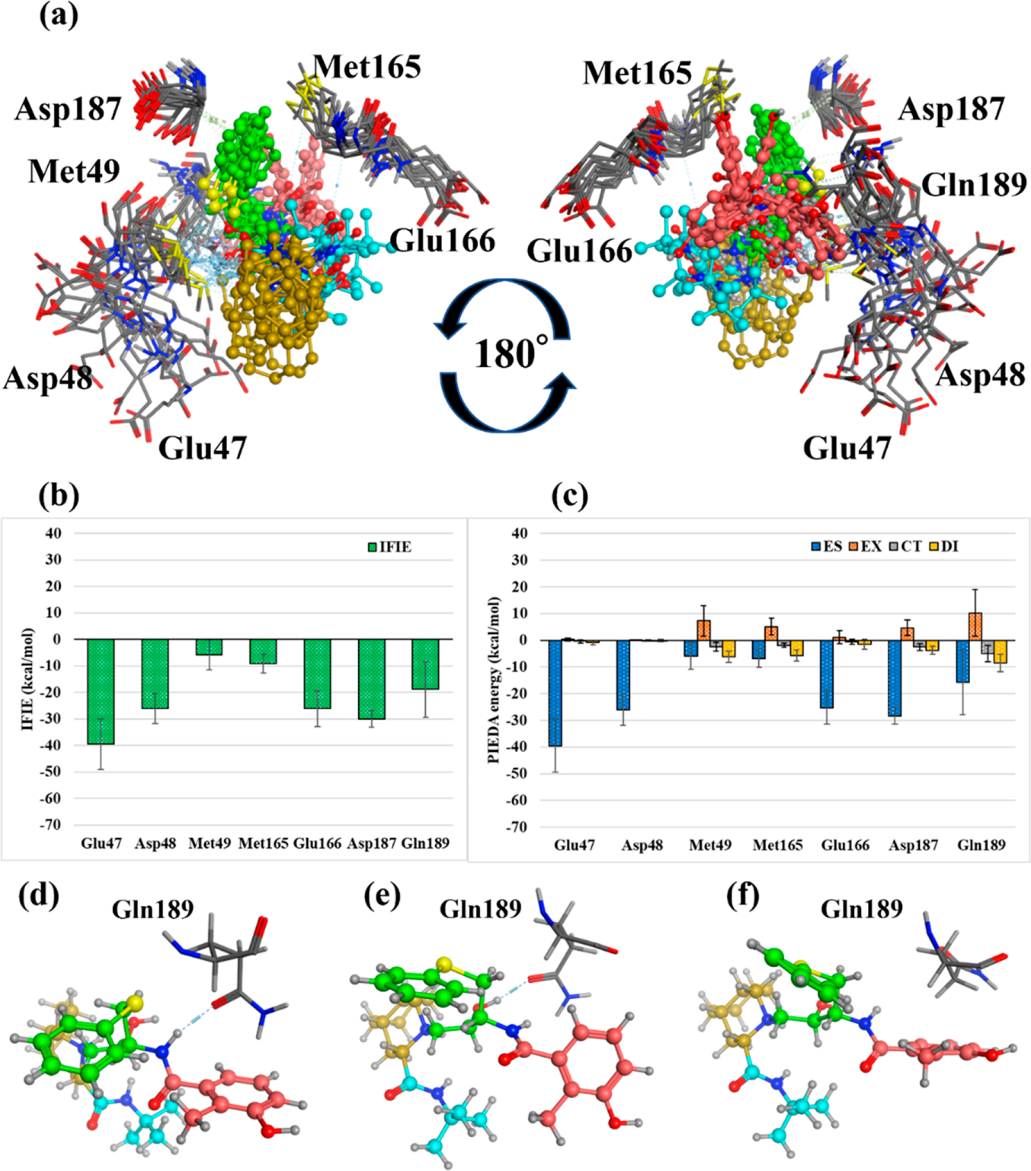
Interactions with the surrounding amino acids in Pose 2. (a) Amino acid residues of main protease interacting with Nelfinavir in Pose 2. The four ligand fragment colors: F1, pink; F2, green; F3, yellow; F4, light blue. (b),(c) Interaction energies between each amino acid residue and NFV: (b) IFIE and (c) PIEDA energies. (d) NH···O hydrogen bond between Gln189 and F1 of NFV (at 55 ns of MD simulation). (e) OH···O hydrogen bond between Gln189 and F2 of NFV (at 75 ns of MD calculation). (f) CH/π interaction between Gln189 and F1 of NFV (at 85 ns of MD simulation).

**Figure 8:**
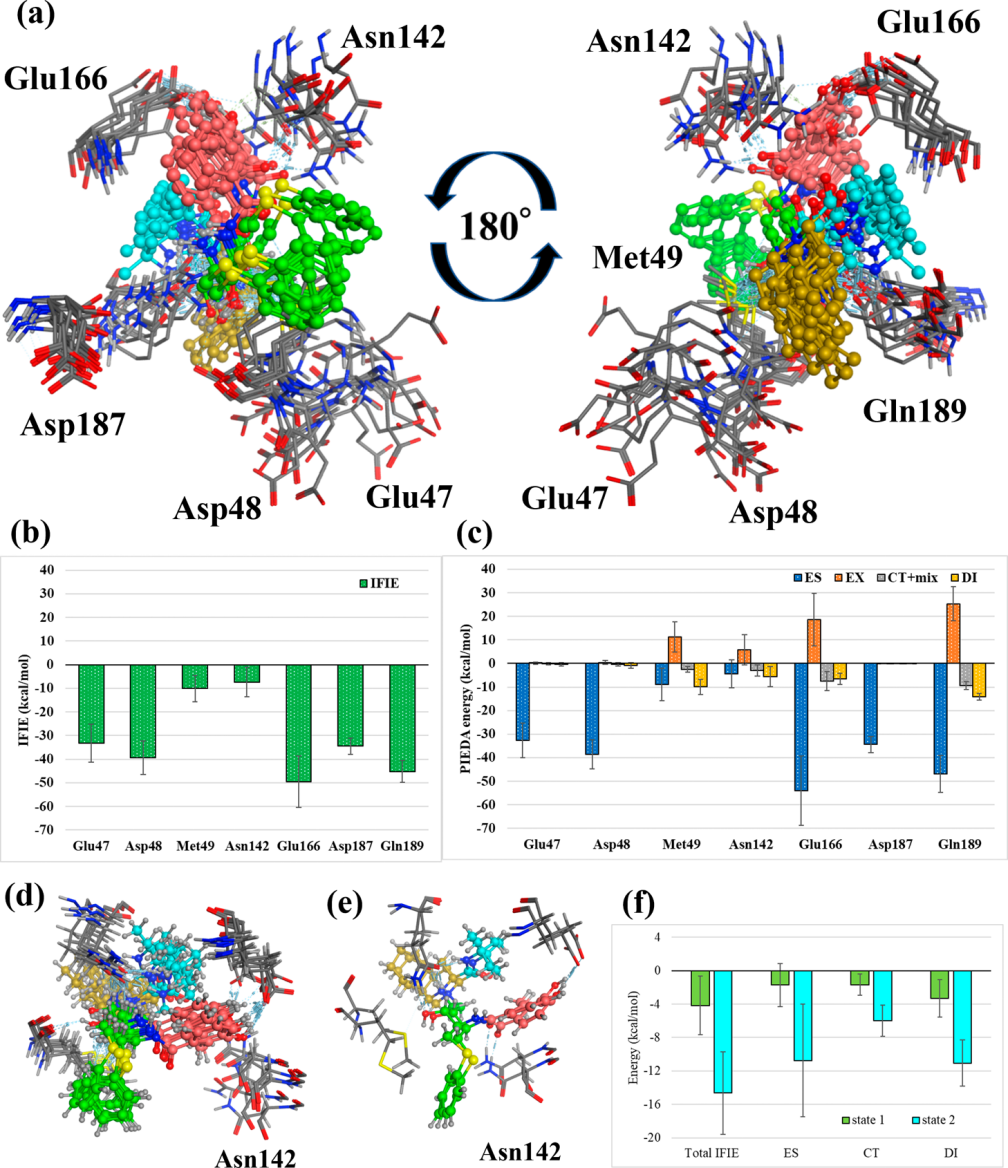
Interactions with the surrounding amino acids in Pose 3. (a) Amino acid residues of main protease interacting with Nelfinavir in Pose 3. The four ligand fragment colors: F1, pink; F2, green; F3, yellow; F4, light blue. (b),(c) Interaction energies between each amino acid residue and NFV: (b) IFIE and (c) PIEDA energies. (d) Structure of Asn142 and NFV from 50 to 85 ns. (State 1). (d) Structure of Asn142 and NFV from 85 to 100 ns. (State 2). (f) IFIE and PIEDA between NFV and Asn142 in State 1 and State 2.

**Figure 9:**
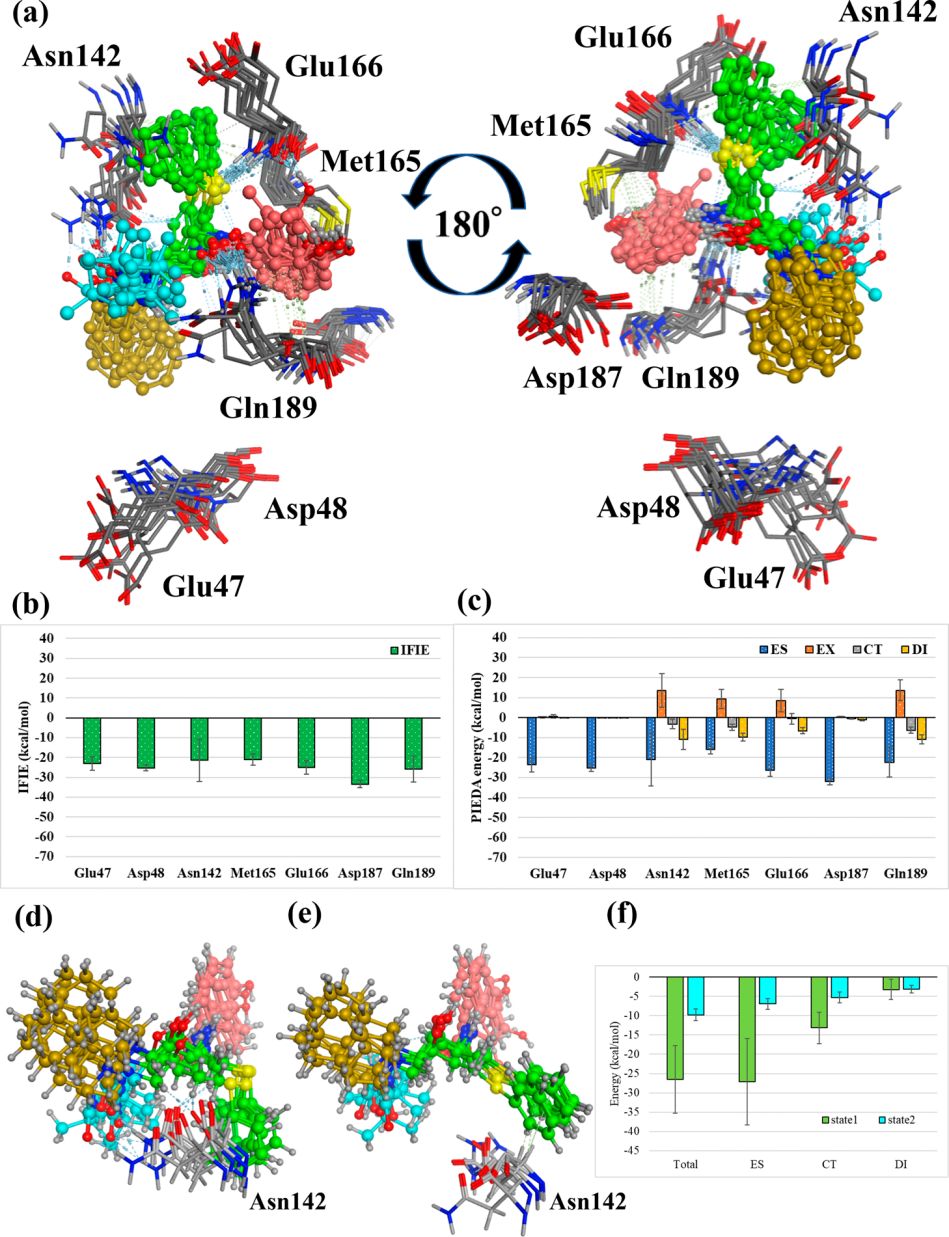
Interactions with the surrounding amino acids in Pose 4. (a) Amino acid residues of main protease interacting with Nelfinavir in Pose 4. The four ligand fragment colors: F1, pink; F2, green; F3, yellow; F4, light blue. (b),(c) Interaction energies between each amino acid residue and NFV: (b) IFIE and (c) PIEDA energies. (d) Structure of NFV and Asn142 in State 1 (50–75 ns) of Figure 5. (e) Structure of NFV and Asn142 in State 2 (80–100 ns) of Figure 5 (f) IFIE and PIEDA between NFV and Asn142 in States 1 and 2.

Figure 6 depicts the amino acid residues of Mpro interacting with NFV in Pose 1. Here, fragments in which the distance between nearest neighbor atoms is within 2.0 times the sum of the van der Waals radius of the atoms are defined as neighboring fragments. Besides the neighboring fragment, only residues with a total IFIE more stable than −15 kcal/mol or a DI term more stable than −5 kcal/mol are illustrated (similar criteria are used in Figures 7–9). Notably, the positive net charge of NFVs overestimates the ES interactions between NFVs and each residue.^52^ Notable PIEDA energy values range from several negative tens of kcal/mol for the ES term; however, even −5 kcal/mol for the DI term is significant. In interactions with the whole NFV, Leu27 had an IFIE of −17.9 ± 8.2 kcal/mol (ES, −18.4 ± 11.0 kcal/mol; CT, −4.5 ± 3.0 kcal/mol; DI, −4.7 ± 1.6 kcal/mol), suggesting hydrogen bonding based on ES and CT energies and CH/π interactions based on the DI energy. Similarly, consideration was made based on each PIEDA component. Glu47, Asp48, and Asp187 showed strong IFIE peaks; however, they were all due to the ES energy. Hence, they are considered electrostatic interactions between fragments that are not in direct contact. Additionally, Asn142 had an IFIE of −10.7 ± 6.6 kcal/mol (ES, −6.8 ± 6.8 kcal/mol; CT, −4.7 ± 2.7 kcal/mol; DI, −8.9 ± 4.0 kcal/mol), suggesting hydrogen bonding based on stabilization of ES and CT energies and CH/π interaction based on stabilization of the DI energy. Moreover, the large standard deviation, particularly in the ES term, suggests that the interaction changes with time in the MD trajectories. Predominantly, Glu166 had an ES energy and strong electrostatic interaction (ES: −34.1 ± 5.7 kcal/mol), and the DI energy was slightly stable (DI: −4.7 ± 2.7 kcal/mol). This suggests CH/π interactions. Furthermore, Gln189 had an IFIE of −14.8 ± 16.3 kcal/mol (ES, −11.5 ± 16.3 kcal/mol; CT, −3.1 ± 3.2 kcal/mol; DI, −6.8 ± 3.8 kcal/mol), suggesting hydrogen bonding based on ES and CT energies and dispersive interactions between hydrophobic functional groups based on the DI energy. The large standard deviation of Gln189 is due to fluctuations in interactions with F3 and F4, which will be described later.

Detailed interactions for each fragment are as follows. For F1, the IFIE with Leu27 was −15.3 ± 7.8 kcal/mol (ES, −15.8 ± 10.6 kcal/mol; CT, −4.5 ± 3.0 kcal/mol; DI, −4.7 ± 1.6 kcal/mol). We observed a hydrogen bond between the OH group of F1 and Leu 27 (the C=O from Leu26 belongs to the Leu27 fragment due to fragmentation rules) and a CH/π interaction between the benzene ring of F1 and the Leu27 side chain. For F2, the IFIE with Glu166 was −6.3 ± 3.7 kcal/mol (ES, −4.8 ± 3.5 kcal/mol; CT, −0.9 ± 1.4 kcal/mol; DI, −4.6 ± 2.8 kcal/mol). The CH/π interaction in the benzene ring of F2 with CH in the side chain of Glu166 was found from the DI energies. The IFIE with Asn142 was −3.2 ± 2.6 kcal/mol (ES, −0.4 ± 2.6 kcal/mol; CT, −3.2 ± 1.5 kcal/mol; DI, −6.7 ± 2.8 kcal/mol), and we observed a CH/π interaction between F2 and the Asn142 side chain. From approximately 95 ns, F2 left its binding site and moved to the solvent-exposed site. The interaction energy from 95–100 ns decreased to approximately 70% of the interaction energy from 50–94 ns. In F3 and F4, F3 showed an IFIE with Gln189 of −12.2 ± 9.6 kcal/mol (ES, −9.9 ± 9.0 kcal/mol; CT, −1.7 ± 1.2 kcal/mol; DI, −3.6 ± 1.4 kcal/mol). F4 showed an IFIE with Gln189 of −2.1 ± 7.3 kcal/mol (ES, −1.6 ± 8.6 kcal/mol; CT, −1.1 ± 1.8 kcal/mol; DI, −2.8 ± 2.0 kcal/mol). From these results, although Gln189 has timing that forms hydrogen bond bridges with N of F3 and N of F4, it failed to maintain this bridge in several sampling structures, as observed by the large standard deviations of IFIE and ES. Notably, the *tert*-butyl group of F4 did not interact.

Therefore, although Pose 1 showed partially strong binding, it is difficult to say that it is an excellent binding pose because F2 could not maintain the interaction and F4 was not used well for binding.

##### 3.3.1.2 Pose 2

Figure 7 shows the amino acid residues of the neighboring fragments interacting with the NFV of Pose 2. Regarding the interaction with the whole NFV, IFIE was strong in Glu47, Asp48, and Glu166, but mostly due to the ES term; hence, it is considered a slightly distant electrostatic interaction. Moreover, Met49 and Met165 showed a hydrophobic interaction due to stabilization of the DI energy. However, since it exists near the ligand, the EX energy was strong, canceling the stabilizing interaction and thereby weakening the IFIE. Asp187 had DI and ES terms, suggesting that it acquires CH/π and ES interactions. The contributions of ES, CT, and DI terms suggest that Gln189 had hydrogen bonds and CH/π interactions. However, considering the large standard deviation, these residues may change their interaction depending on the timing of sampling from the MD.

Detailed interactions for each fragment are as follows. In F1, IFIE with Gln189 was −5.2 ± 5.0 kcal/mol (ES, −4.3 ± 6.4 kcal/mol; CT, −2.4 ± 2.3 kcal/mol; DI, −3.9 ± 3.0 kcal/mol). We observed a hydrogen bond between the N of F1 and the carbonyl of the Gln189 side chain and a CH/π bond between the benzene ring of F1 and the CH of the Gln189 side chain (Figure 7d,f). However, the standard deviation of each component was large; hence, their interactions were not necessarily retained. In F2, IFIE with Gln189 was −9.0 ± 4.1 kcal/mol (ES, −6.8 ± 5.0 kcal/mol; CT, −2.6 ± 1.5 kcal/mol; DI, −4.5 ± 1.7 kcal/mol). We observed a hydrogen bond between the OH group of F2 and the carbonyl of Gln189; however, the interaction was not preserved because of the large standard deviations. Moreover, we suggested the formation of hydrophobic interactions between CHs of F2 and Gln189 side chains (Figure 7e). Additionally, the DI energy with Met165 was −4.0 ± 1.5 kcal/mol, and the DI energy with Asp187 was −3.7 ± 1.5 kcal/mol, suggesting the formation of a CH/π bond. F3 and Met49 had a DI energy of −2.6 ± 1.5 kcal/mol, suggesting a hydrophobic interaction; however, we observed no other interacting residues with IFIE > −5 kcal/mol. Similarly, in F4, no interacting residues with IFIE > −5 kcal/mol were identified. Therefore, although Pose 2 formed partially strong interactions, F3 and F4 were not used well for binding, suggesting that it is not a good binding pose.

##### 3.3.1.3 Pose 3

Figure 8 depicts the amino acid residues of the neighboring fragments interacting with the NFV of Pose 3. Since this pose had a stronger ES term than other poses, the energy for each amino acid had a stronger ES term. Regarding the interaction with the whole NFV, Glu47, Asp48, and Asp187 had mostly ES terms; however, Glu166 and Gln189 had CT and ES terms, suggesting that they form hydrogen bonds. Although Met49 had a total IFIE weaker than those of other residues, it had a DI term of −10.0 ± 3.3 kcal/mol, suggesting a strong hydrophobic interaction. Lastly, although Asn142 did not show a strong interaction overall, it acquired a strong interaction depending on the time of MD sampling.

Detailed interactions for each fragment are as follows. In F1, the IFIE with Glu166 was −24.5 ± 9.7 kcal/mol (ES, −29.9 ± 13.7 kcal/mol; CT, −7.1 ± 4.0 kcal/mol; DI, −5.1 ± 2.0 kcal/mol). We observed a hydrogen bond between the hydroxy group of F1 and the side chain carbonyl group of Glu166 and a CH/π bond between the benzene ring of F1 and the CH of the side chain of Glu166. Moreover, F2 had a DI energy of −6.2 ± 2.1 kcal/mol with Met49, and the benzene ring of F2 had a CH/π bond with the side chain of Met49. F3 interacted with Gln189 at −29.2 ± 3.1 kcal/mol (ES, −28.8 ± 5.0 kcal/mol; CT, −5.1 ± 1.2 kcal/mol; DI, −6.1 ± 0.8 kcal/mol). Besides the hydrophobic interactions with the hydrocarbon moiety of F3, the N of F3 and the side-chain carbonyl of Gln189 formed hydrogen bonds. Furthermore, F4 interacted with Gln189 at −12.5 ± 1.4 kcal/mol (ES, −15.5 ± 2.7 kcal/mol; CT, −2.5 ± 1.0 kcal/mol). Therefore, N of F4 is hydrogen-bonded to the carbonyl group of the Gln189 side chain, similar to F3. Notably, the *tert*-butyl group of F4 did not interact.

In Figure 5, we considered that the state of ligands in MD was divided into two types, 50–85 ns (State 1) and 85–100 ns (State 2). The conformation change during MD simulation was discovered because the FMO calculations after MD showed different interaction energies with some amino acids compared to before MD. The difference of states was mainly due to structural changes in F2. Met49 maintained the binding by moving cooperatively with F2. However, Asn142 changed its structure in each state; therefore, the interaction differed (Figure 8d–f). Moreover, the total IFIE (*ΔE*^*int*^) was more than three times higher in State 2 than in State 1, and the interaction was enhanced in State 2 and ES, CT, and DI. This is because in State 2, the carbonyl of F1 and N of Asn142 formed a new hydrogen bond, and the benzene ring of F1 and the main chain carbonyl of Leu141 (Asn142 C=O) formed a new π/π interaction.

Therefore, Pose 3 could use all fragments for binding, and its binding energy (Δ*E*^*int*^) suggests that it is the strongest among the four poses. Moreover, Δ*E*^*int*^ increased from −203.2 ± 17.2 to −232.5 ± 13.2 kcal/mol with the change from State 1 to State 2. This observes that State 2 is stable in terms of binding energy. Consequently, functional groups that have fluctuating interactions with specific residues, such as Asn142, are candidates for molecular optimization, and it is expected that converting them to stable interactions will improve binding affinity.

##### 3.3.1.4 Pose 4

Figure 9 shows the amino acid residues of the neighboring fragments interacting with the NFV of Pose 4. Regarding the interaction with the whole NFV, Glu47, Asp48, and Asp187 had strong IFIEs, which were mostly electrostatic interactions due to the ES term. Moreover, Met165, Glu166, and Gln189 had DI and ES terms attributed to the CH/π interaction with the ligand. Asn142 had ES, CT, and DI terms, suggesting hydrogen-bonding and CH/π interactions. However, since standard deviations were large, the structural changes due to MD were significant, as with Pose 3.

Detailed interactions for each fragment are as follows. F1 interacted with Gln189 at −14.4 ± 4.8 kcal/mol (ES, −12.6 ± 6.3 kcal/mol; CT, −5.2 ± 1.4 kcal/mol, DI, −8.7 ± 2.0 kcal/mol), suggesting hydrogen and CH/π bonding constituted by NH/O hydrogen bonds between the carbonyl group of F1 and the Gln189 side chain and CH/π bonds between the benzene ring of F1 and the Gln189 side chain. Additionally, FI interacted with Met165 at −6.4 ± 1.4 kcal/mol in the DI term, suggesting the formation of a CH/π bond between the benzene ring of F1 and the side chain of Met165. F2 formed a CH/π bond between its benzene ring and the side chain of Asn142 since the DI energy with Asn142 was −6.9 ± 2.3 kcal/mol. Additionally, the DI energy of F2 and Glu166 was −5.9 ± 1.5 kcal/mol, suggesting that the benzene ring of F2 formed a CH/π bond with the side chain of Glu166. F2 underwent structural changes at 80 ns (Figure 9d,e) but retained the CH/π bond with Asn142. F3 had electrostatic interactions with negatively charged residues, such as Glu47 and Asp48, due to the positive charge of N; however, no hydrogen-bonding or CH/π interactions was observed. Lastly, F4 formed a hydrogen bond with Asn142 in the structure from 50–75 ns (State 1) (IFIE, −11.9 ± 4.8 kcal/mol; ES, −12.6 ± 6.0 kcal/mol; CT, −2.6 ± 1.2 kcal/mol). However, the structure after 80 ns lost hydrogen bonding (IFIE, −0.1 ± 1.3 kcal/mol; ES, 0.2 ± 1.3 kcal/mol; CT, −0.0 ± 0.1 kcal/mol). Notably, the *tert*-butyl group did not interact in Pose 4.

Therefore, Pose 4 could use all fragments for binding, and its binding energy (Δ*E*^*int*^) was stronger than those of Poses 1 and 2, which only partially interacted. Regarding Asn142, confirming the interaction observed from the whole ligand (Figure 9f), State 1 acquired more than twice the interaction compared with State 2. Additionally, the interaction was enhanced in ES and CT due to structural changes in F2, F4, and Asn142 at 80 ns.

The *tert*-butyl group of NFV, which was not found to interact with Mpro through the four poses, was compared with HIV-1 protease, the original target of NFV. In the complex structure of HIV-1 protease and NFV (PDBID: 3EL5), the binding interaction energy Δ*E*^*int*(*static*)^ was −155 kcal/mol, slightly more stable than Pose 2. However, the *tert*-butyl group, which did not interact with Mpro, interacted strongly in the DI term with the surrounding hydrophobic amino acid residues (Ala28, Asp29, Asp30, Val32, Ile47, etc.) in the HIV-1 protease (Figure S5). In other words, all functional groups were utilized in the interaction with the original target protein. Thus, identifying such functional groups with less robust interactions is a candidate for functional group optimization and is an important suggestion for drug discovery using drug repositioning.

#### 3.3.2 Ligand-Binding Energy

Table 1 presents the results (Δ*E*^*int*^) of calculating the average value and standard deviation of total IFIE from the FMO calculation results of each of the 100 structures for Poses 1–4. Comparing the dynamically averaged interaction energy Δ*E*^*int*^ with the static interaction energy Δ*E*^*int*(*static*)^ showed that Pose 3 was consistently the most stable. Moreover, Δ*E*^*int*^ showed that Pose 4 was the second most stable structure, followed by Pose 1, and the stability was reversed from that of the static state. This is because, although Pose 1 had two hydrogen bonds and Pose 4 had one hydrogen bond in the static structure, and both poses had three bonds in the dynamic structure, the hydrogen bond dependence on the time was not maintained in Pose 1 and Pose 4 was in a stronger binding state. In the DI term in Δ*E*^*int*(*static*)^, Poses 1 and 2 were more stable than Poses 3 and 4 by approximately −20 kcal/mol. Conversely, in the DI term in Δ*E*^*int*^, Poses 3 and 4 showed stable values ranging from −5 to +10 kcal/mol. Furthermore, in the static structure, there are four CH/π interactions in Pose 1, three in Pose 2, three in Pose 3, and three in Pose 4. However, in the dynamical structure, there are three CH/π interactions in Pose 1, two in Pose 2, two in Pose 3, and five in Pose 4. The number of interactions decreased in Poses 1 and 2 and increased in Pose 4, explaining why the DI term became more stable than those in Pose 1 and Pose 2. Moreover, Pose 3 had few CH/π interactions; however, its hydrophobic interactions with Gln189 and others stabilized the DI term. To perform an experimental validation of the prediction ability of this method, we obtained Δ*E*^*int*^ for LPV using the same procedure and compared to NFV. As shown in Tables S2 and S3, the Δ*E*^*int*^ of these NFV (−136 to −212 kcal/mol) were generally more stable than those of LPV (−64 to −100 kcal/mol), consistent with the order of IC_50_ values (NFV, 0.77 μM; LPV, 3.07 μM).^20^

Next, the deformation energy (Δ*E*_*lig*_^*def*^) of the ligands of each pose and the binding energy, Δ*E*^*int*^ + Δ*E*_*lig*_^*def*^, are presented in Table 1. Poses 1–3 had similar binding energies, and Pose 4 had a stronger deformation energy than other poses. This is because Pose 4 has a wider shape than the other poses and is connected distortedly. Additionally, Δ*E*^*int*^ + Δ*E*_*lig*_^*def*^ values were 3, 4, 1, and 2 in descending order. Since the deformation energy is the same as the magnitude relationship of Δ*E*^*int*^, it has little effect on the difference in Δ*E*^*int*^.

**Figure 10:**
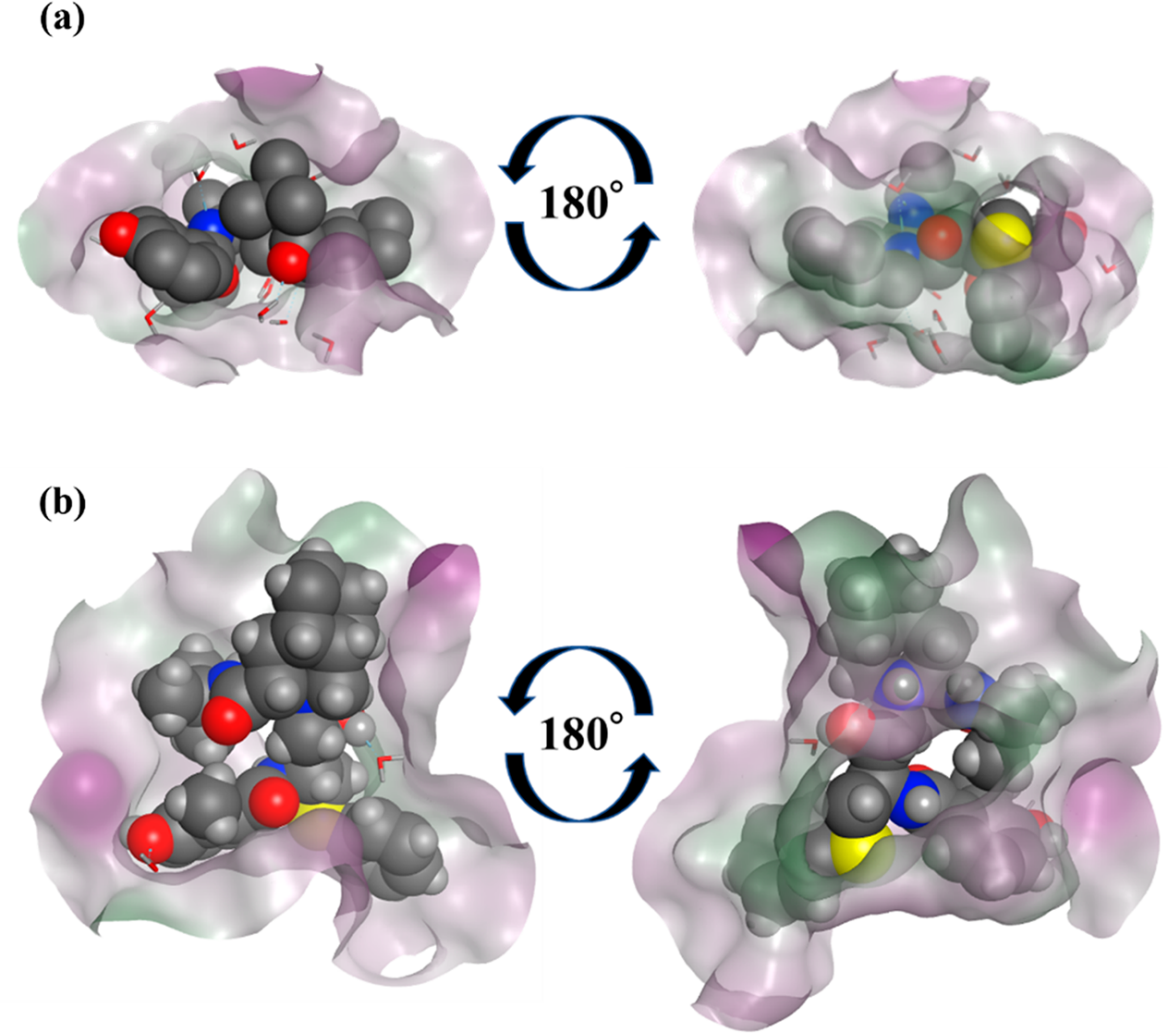
Water molecules in the ligand-binding pocket. (a) Structure of Pose 2 at 100 ns. Water molecules can be seen between the ligand and the protein. (b) Structure of Pose 3 at 100 ns, with almost no water molecules between the ligand and the protein.

Furthermore, the desolvation energy (Δ*E*^*sol*^) was determined. Using the method defined in Section 2.5, 25 amino acids within a 4 Å average distance from ligands were selected. The solvation energies of Poses 3 and 4 were approximately 120 kcal/mol, twice as much as those of Poses 1 and 2 (approximately 60 kcal/mol) (Table 1). Figure 10 panels (a) and (b) show the actual water molecules around ligands and binding sites in Poses 2 and 3. In Pose 2, the water molecules were trapped between the ligand and protein in the pocket (Figure 10a). However, in Pose 3, the ligand was tightly bound to the protein, and almost no water molecules entered the binding site (Figure 10b). Notably, water molecules in the ligand-binding pocket reduce direct interactions between the ligand and amino acid residues at the binding site. Therefore, Δ*E*^*int*^ was less stabilized and the desolvation energy (Δ*E*^*sol*^) was weak because less water was displaced by binding. Specifically, in Poses 1 and 2, many water molecules remained at the binding sites; hence, Δ*E*^*int*^ was less stabilized and Δ*E*^*sol*^ was weak. However, in Poses 3 and 4, Δ*E*^*int*^ was greatly stabilized and Δ*E*^*sol*^ was strong, indicating that most water molecules at the binding sites were excluded. In fact, the average number of water molecules within 3.4 Å of Ligand was 26.1 ± 9.6 for Pose 1, 30.7 ± 9.2 for Pose 2, 21.1 ± 7.4 for Pose 3, and 21.6 ± 10.2 for Pose 4. Although this includes the number of water molecules in the ligand site exposed in bulk water, the difference corresponds to the difference in the number of water molecules in the binding sites of NFV and Mpro: Pose 1 and Pose 2 have 5–9 more water molecules in the binding site than Pose 3 and Pose 4, which may result in weaker desolvation effects. Moreover, two water molecules in the catalytic dyad that are conserved among several crystal structures^67^ were observed also in the MD calculations of this study: both were conserved in Pose 1 and Pose 4, and only one was conserved in Poses 2 and 3. This is consistent with the Δ*E*^*sol*^ trend in Table 1. Such direct interactions with water molecules cannot be handled using the FMO-PB calculation^36^ or the continuum model PCM,^68^ and the results of this study were obtained only by explicitly treating water molecules. Δ*E*_bind_ that has ultimately been obtained is presented in Table 1. In Δ*E*_bind_, we suggested that 3, 1, 2, and 4 were the stable binding poses in order of decreasing stability. Additionally, Δ*E*_bind_ in Poses 3, 1, and 2 was negative but positive in Pose 4 and could not be stabilized by binding. Considering the difference between Δ*E*_*bind*_ and Δ*E*^*int*^, we can understand the stabilizing and destabilizing factors in the coupling energy calculation. Pose 4 shows that Δ*E*^*int*^ stabilizes all four ligand fragments. However, as shown in eq 3, Δ*E*_*bind*_ is the sum of Δ*E*^*int*^, Δ*E*_*lig*_^*def*^, and Δ*E*^*sol*^. Δ*E*_*lig*_^*def*^ is the strain energy of the ligand that is deformed by binding, and Δ*E*^*sol*^ is the energy representing the effect of pulling off the hydration water in binding, both of which are positive (unstable) values. Pose 4 has the second strongest Δ*E*^*sol*^ after Pose 3 while Δ*E*^*int*^ is less stable than the other poses, suggesting greater destabilization due to desolvation. Thus, Δ*E*_*bind*_ in Pose 4 is positive, suggesting that it may be difficult to bind Mpro. In other words, binding is determined by the balance between stabilization by Δ*E*^*int*^ and destabilization by Δ*E*_*lig*_^*def*^ and Δ*E*^*sol*^.

Although the binding energy values differed depending on the pose, some amino acids interacting with the ligand were common in the four binding poses. Glu47, Asp48, and Asp187 acquired electrostatic interactions in all of the binding poses. Similarly, Glu166 acquired electrostatic interactions in all binding poses and hydrogen bonds or CH/π interactions, depending on the pose. Moreover, Gln189 had a hydrogen bond and CH/π interaction in all poses; however, it fluctuated greatly depending on the pose. These are consistent with known MD results^9,69^ that Glu166, Asp187, Gln189, etc. are important ligand-binding sites. The energies between these residues and NFVs and distances between their nearest neighboring atoms are listed in Table S4. Asp48 and Asp187 are not necessarily close to NFVs, mainly due to ES interactions. Glu166 and Gln189, which have hydrogen bond and CH/π interactions, are close in distance for all poses. However, the strength of the stabilization energy is not determined by the distance alone, and it is also important to observe the standard deviation: for example, in the comparison between Pose 2 and Pose 4, the distances to Glu47, Asp48, and Glu166 are closer but less stabilized in Pose 4. In addition, Gln189 has a strong interaction with Pose 3, where the standard deviation of the distance is particularly small, but it has a weak interaction with Pose 1 and Pose 2, where the standard deviation of the distance is relatively large. Asn142 was also identified as a residue with a hydrogen bond or CH/π interaction in all poses excluding Pose 2; however, it was a fluctuating interacting residue in all binding poses of Poses 1, 3, and 4. Furthermore, Met49 and Met165 showed a strong interaction in two poses and Leu27 showed a strong interaction in one pose. Gln189 maintained hydrogen bonds with the peptide-like backbones of F3 and F4 in Pose 3 and F1 in Pose 4. In contrast, in other poses, there were times when the nitrogen of the peptide-like backbone of F3 and F4 in Pose 1 and the OH group of the peptide-like backbone of F1 and F2 in Pose 2 made a hydrogen bond bridge with Gln189; however, several of the sampling structures failed to retain the bond. Similarly, Asn142 was hydrogen-bonded with the peptide-like backbone of F1 in Pose 3 and that of F4 in Pose 4. The stability of Δ*E*^*int*^ in Poses 3 and 4 suggest that strong hydrogen bonding with the peptide-like backbone is one of the factors responsible for the stability of binding to Mpro.

For compounds that inhibit the Mpro, described in a study^31^ that comprehensively analyzed the interaction of 110 complex ligand structures registered in FMODB, CH/π interactions with Asn142, hydrogen bonds with the main chain of Glu166, and CH/π interactions with the β-carbon are important. Furthermore, a study^10^ on the crystal structure of Ensitrelvir (PDBID: 7VTH), which targets the Mpro, reported that Ensitrelvir hydrogen-bonds with the main chain NH of Glu166. Moreover, Asn142, identified as a residue with a fluctuating interaction in this study, had a missing atom in the side chain in the crystal structure of Ensitrelvir. Therefore, in the future, better binding properties can be acquired by designing compounds with stable interactions with Asn142. Although the results obtained using our method do not perfectly predict the binding pose itself, we were able to identify interacting residues that are important for ligand recognition.

## 4 Conclusions

We performed docking, MD, and FMO (MD + FMO) calculations to estimate the binding poses and clarify the binding properties of the Mpro and NFV complex, a drug repositioning candidate whose crystal structure has not been solved. Furthermore, we identified the intermolecular interactions between the NFV functional groups and Mpro residues that are important for ligand recognition. We ranked each pose by evaluating the binding energy. In addition to the intermolecular interaction, the desolvation effect was important in this procedure. The exclusion of water molecules from the ligand-binding pocket is believed to be important for Mpro binding to NFV. The calculation of the desolvation energy by explicit solvent consumes enormous computational resources. However, the use of a Fugaku supercomputer has made it possible. In the analysis of the intermolecular interaction focusing on the NFV side, the following interactions were crucial in all poses: the CH/π and π/π interactions between the π electrons of the benzene rings of F1 and F2 and surrounding residues and hydrogen-bonding interactions with the OH group of F1 and surrounding residues. Moreover, the *tert*-butyl group of F4 was not involved in the binding; hence, it was considered unimportant for binding to the Mpro. However, F4 was an important functional group in binding to HIV-1 protease—the original target of NFV. Therefore, functional groups that became less important due to drug repositioning could be identified using our method. Moreover, the hydrogen bond between the peptide-like backbone and Mpro was considered important. This backbone portion formed hydrogen bonds with Gln189 in Poses 3 and 4. However, in Poses 1 and 2, hydrogen bonds were rarely formed at this site. Therefore, we inferred that the strong hydrogen bond at the peptide-like site is a factor in the binding stability of Mpro and NFV.

Additionally, on the Mpro side, amino acid residues important for binding (Glu47, Asp48, Glu166, Asp187, and Gln189), common to all binding poses, could be identified. These amino acid residues were considered important in drug design. Furthermore, we suggested that it is important to have hydrogen bonds with peptide-like backbones and multiple π-rich functional groups, such as benzene rings, in interactions with these residues, particularly for NFV inhibition.

Our “MD+FMO” approach can predict the local stable structure of protein–ligand complexes considering the conformational fluctuations in the solvent and identifying important intermolecular interactions in the complexes. Quantitative interaction energies based on quantum chemical calculations generate ideas for subsequent molecular design. Our approach provides a new guideline for structure-based drug design starting from a candidate compound whose complex crystal structure has not been obtained. On the other hand, for a more precise prediction in the future, it is comprehensively necessary to consider stereo isomers of ligand, ligand conformational searches, use of ensemble docking, and use of multiple template structures. In addition, a consideration of part of the binding free energy is also an issue to be addressed in the future.

When applying the conventional static FMO to drug design, the FMO method is useful for lead optimization projects where the protein is reasonably stiff.^70^ In the present study, even when there are multiple conformations of the interaction between the ligand and the residues in the pocket, the MD+FMO method further enables us to identify residues with altered interactions and quantify the energetic impact of their fluctuations on the binding stability. This will provide a new guide for medicinal chemists and may be useful in the development of therapeutic agents for the treatment of COVID-19 and other diseases.

## Data Availability

All structure files and a set of input/output files used for FMO calculations are available at the FMODB (https://drugdesign.riken.jp/FMODB/); FMODBIDs are listed in Table S5. Simple data analysis can be performed using the FMODB web interface, and detailed analysis can be performed using the BioStation Viewer software (https://fmodd.jp/biostationviewer-dl/). FMO Software ABINIT-MP has been preinstalled on high-performance computing infrastructure machines (https://www.hpci-office.jp/pages/e_appli_abinit-mp).

## Supporting Information Available

Six structures of Mpro and lopinavir selected based on docking and FMO scoring; amino acids for which desolvation energies are calculated; FMO calculation results for 30 structures obtained by docking; interaction analysis of NLF-like compounds and Mpro by FMO; PIEDA for four docking poses; interaction involving the *tert*-butyl group of NLF; FMO calculation results for 15 structures obtained by docking in Lopinavir and Mpro; interaction energies of each docking pose in Lopinavir; distances and energies (Δ*E*^*int*^) between NFV and amino acid residues important for binding that are common to all binding poses; list of FMODB IDs of FMO calculation results for each MD sampling structure (PDF)
