# Supplementary material for: Prediction of Binding Pose and Affinity of Nelfinavir, a SARS-CoV-2 Main Protease Repositioned Drug, by Combining Docking, Molecular Dynamics, and Fragment Molecular Orbital Calculations
Source: J Phys Chem B. 2024 Mar 4;128(10):2249–65. doi: 10.1021/acs.jpcb.3c05564 (PMC10946393; doi:10.1021/acs.jpcb.3c05564)
Supplement: Supplementary file 1 — jp3c05564_si_001.pdf [file jp3c05564_si_001.pdf]

Supporting Information for: Prediction of Binding Pose and Affinity of Nelfinavir, a SARS-CoV-2 Main Protease Repositioned Drug, by Combining Docking, Molecular Dynamics, and Fragment Molecular Orbital Calculations

Yuma Handa[1,2], Koji Okuwaki[1,3], Yusuke Kawashima[1], Ryo Hatada[3], Yuji Mochizuki[3,4], Yuto Komeiji[2,3,5,7], Shigenori Tanaka[6], Takayuki Furuishi[1], Etsuo Yonemochi[1], Teruki Honma[7], Kaori Fukuzawa[1,2,8*]

1. Department of Physical Chemistry, School of Pharmacy and Pharmaceutical Sciences, Hoshi University, 2-4-41 Ebara, Shinagawa-ku, Tokyo 142-8501, Japan
2. Graduate School of Pharmaceutical Sciences, Osaka University, 1-6 Yamadaoka, Suita, Osaka 565-0871, Japan
3. Department of Chemistry and Research Center for Smart Molecules, Faculty of Science, Rikkyo University, 3-34-1 Nishi-ikebukuro, Toshima-ku, Tokyo 171-8501, Japan
4. Institute of Industrial Science, University of Tokyo, 4-6-1 Komaba, Meguro-ku, Tokyo 153-8505, Japan
5. Health and Medical Research Institute, AIST, Tsukuba Central 6, Tsukuba, Ibaraki 305-8566, Japan
6. Graduate School of System Informatics, Department of Computational Science, Kobe University, 1-1 Rokkodai, Nada-ku, Kobe 657-8501, Japan
7. RIKEN Center for Biosystems Dynamics Research, 1-7-22 Suehiro-cho, Tsurumi-ku, Yokohama, Kanagawa, 230-0045, Japan
8. Department of Biomolecular Engineering, Graduate School of Engineering, Tohoku University, 6-6-11 Aoba, Aramaki, Aoba-ku, Sendai 980-8579, Japan

*Corresponding author: Kaori Fukuzawa (fukuzawa-k@phs.osaka-u.ac.jp)

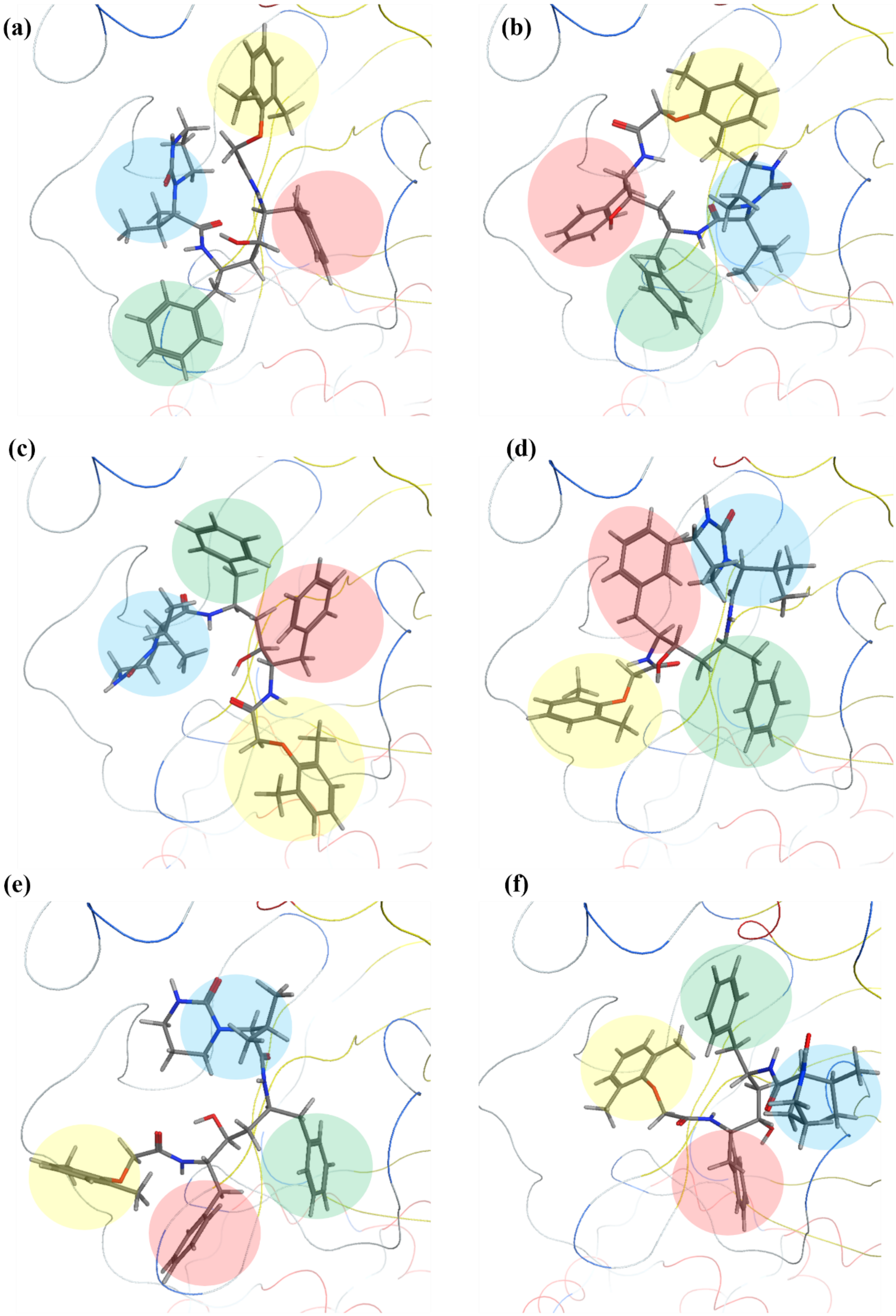

Figure S1 Six structures of Mpro and lopinavir selected based on docking and FMO scoring.
(a) Pose 1, (b) Pose 2, (c) Pose 3, (d) Pose 4, (e) Pose 5, (f) Pose 6.

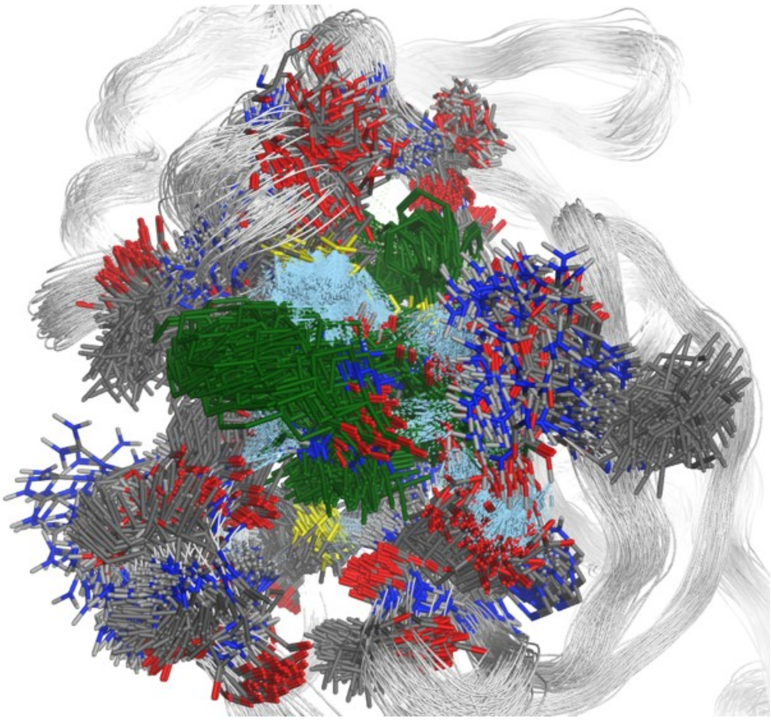

Figure S2 Amino acids for which desolvation energies are calculated: Thr25, Thr26, Leu27, His41, Ser46, Met49, Leu50, Leu141, Asn142, Gly143, Ser144, Cys145, His163, His164, Met165, Glu166, Leu167, His172, Phe181, Asp187, Arg188, Gln189, Thr190, Ala191, Gln192. The figure shows the superposition structure for 50-100 ns in Pose 3. (Green; NLF)

Table S1 FMO calculation results for 30 structures of Mpro-Nelfinavir comlex obtained by docking (in kcal/mol), sorted by FMO score ($\Delta E^{int(static)}$). The four structures with the top FMO scores were selected as Poses 1−4.

docking structure number	docking score	ES	EX	CT + mix	DI	$\Delta E^{int(static)}$ (Total IFIE)
No.7 (Pose 3)	-9.07	-139.5	63.2	-33.1	-83.1	-192.6
No.1 (Pose 1)	-9.70	-150.4	105.1	-35.0	-103.5	-183.7
No.11 (Pose 4)	-9.00	-145.9	89.9	-33.4	-84.9	-174.3
No.2 (Pose 2)	-9.63	-135.1	118.1	-24.4	-105.6	-147.1
No.8	-9.07	-113.0	82.0	-30.3	-84.7	-146.0
No.3	-9.58	-116.6	96.0	-32.8	-91.2	-144.6
No.4	-9.40	-118.1	92.2	-28.0	-90.1	-144.0
No.5	-9.35	-108.9	82.1	-27.9	-88.3	-142.9
No.6	-9.24	-101.0	72.7	-27.5	-81.9	-137.6
No.19	-8.76	-98.4	59.9	-25.4	-64.5	-128.4
No.10	-9.01	-96.5	67.3	-24.7	-74.4	-128.3
No.9	-9.03	-97.3	70.1	-25.1	-75.3	-127.6
No.17	-8.79	-91.2	58.8	-24.9	-69.9	-127.2
No.15	-8.86	-95.0	60.7	-24.3	-66.4	-125.1
No.23	-8.65	-92.3	57.3	-21.1	-66.2	-122.3
No.14	-8.91	-91.5	58.4	-22.1	-66.5	-121.7
No.12	-8.94	-90.3	60.2	-21.3	-69.1	-120.4
No.18	-8.78	-90.1	57.1	-22.1	-64.9	-120.1
No.22	-8.65	-89.3	55.4	-20.8	-62.6	-117.3
No.13	-8.92	-87.1	57.3	-20.2	-67.2	-117.2
No.20	-8.75	-86.3	53.0	-19.2	-60.7	-113.3
No.24	-8.59	-84.6	52.5	-19.5	-60.9	-112.6
No.21	-8.67	-84.8	53.2	-19.1	-60.1	-111.8
No.16	-8.79	-84.8	51.6	-18.6	-59.0	-110.8
No.26	-8.52	-84.1	50.8	-15.9	-60.9	-110.1
No.25	-8.58	-83.8	51.6	-16.7	-60.9	-109.8
No.28	-8.47	-78.8	44.2	-14.3	-59.9	-108.8
No.27	-8.49	-79.6	47.1	-17.1	-58.9	-108.6
No.29	-8.46	-78.9	45.2	-15.4	-57.8	-106.9
No.30	-8.46	-77.8	43.2	-14.0	-57.2	-105.8

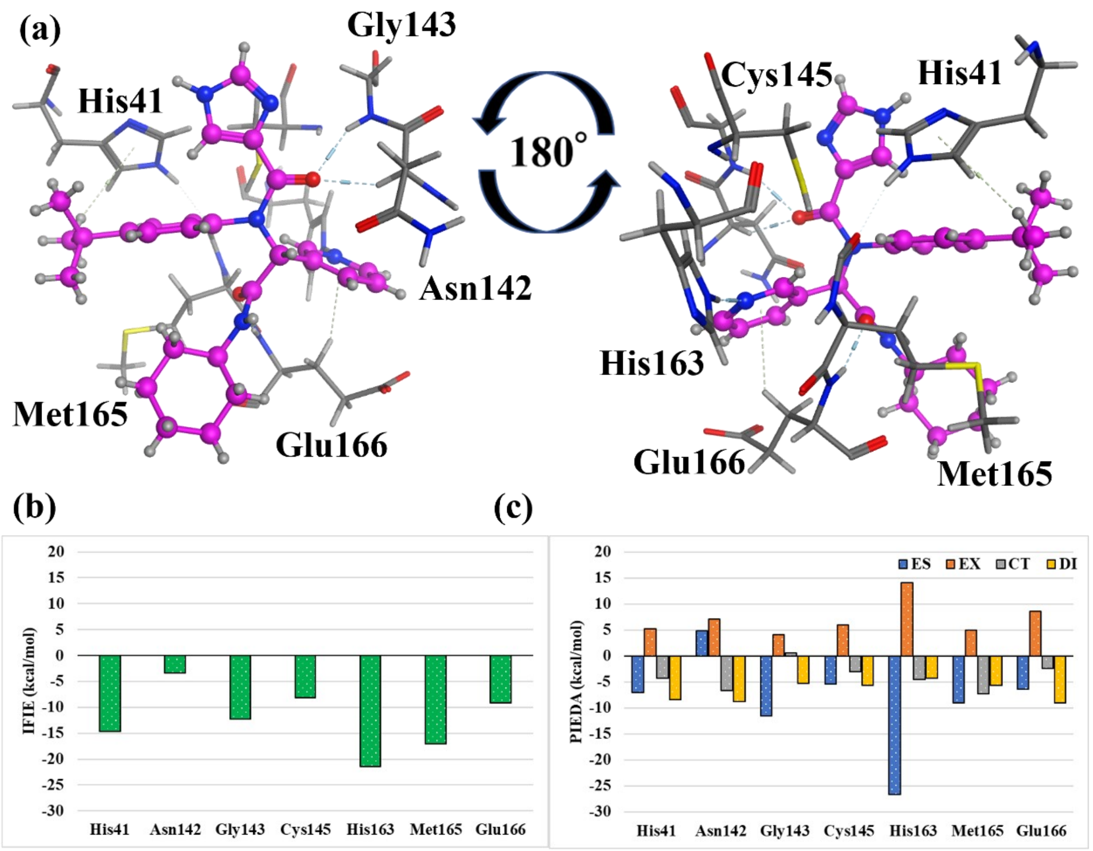

(d)

ES	EX	CT + mix	DI	$\Delta E^{int(static)}$ (Total IFIE)
-95.3	63.6	-38.9	-74.4	-144.9

Figure S3 Interaction analysis of NLF-like compounds and Mpro by FMO. (a) Amino acid residues of Main Protease interacting with NLF-like compounds (PDBID: 6w63). (b)(c) Interaction energies between each amino acid residue and NLF-like compounds; (b) IFIE and (c) PIEDA energies. (d) Total IFIE (Sum of interaction energy) and each PIEDA energy (in kcal/mol).

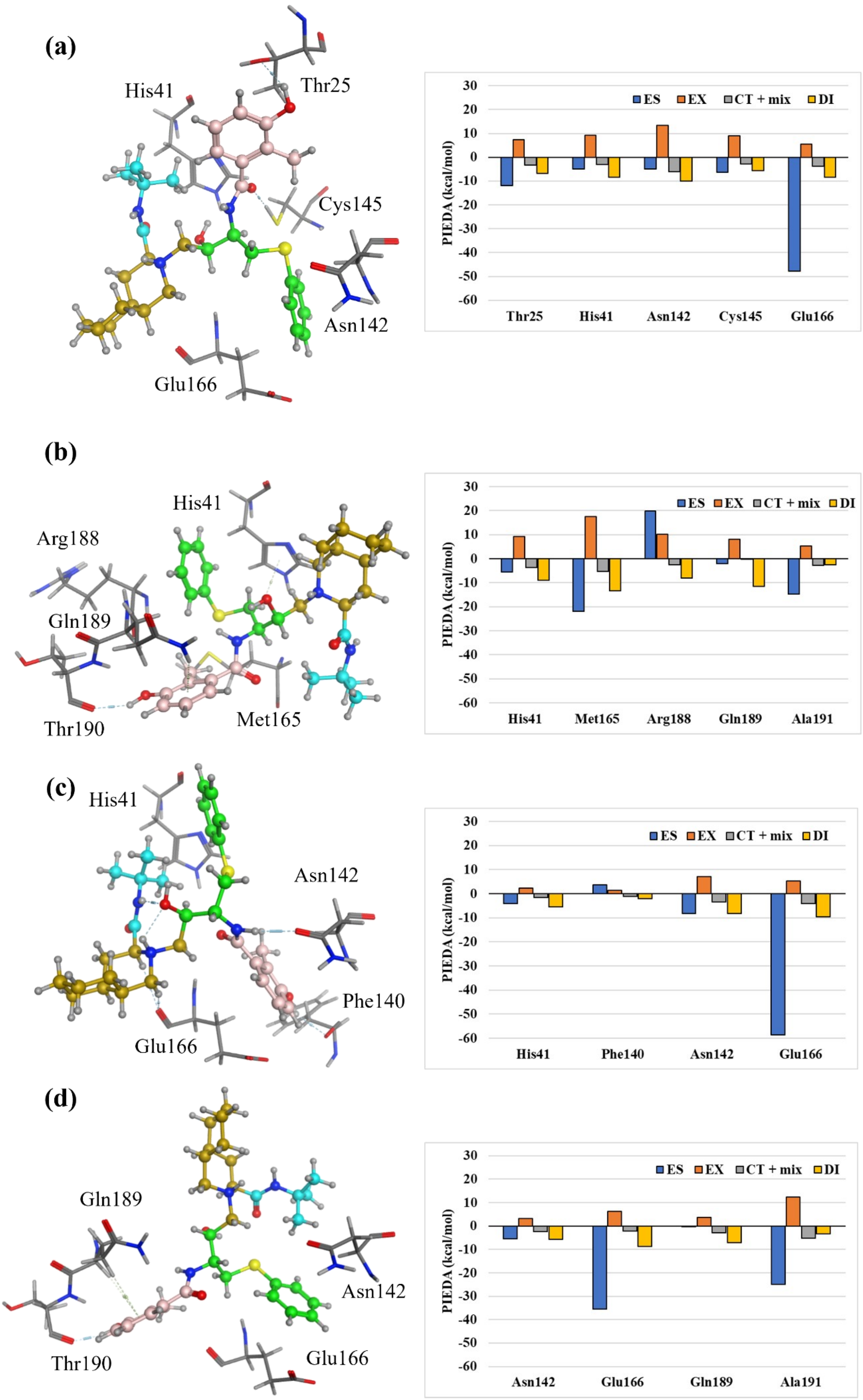

Figure S4 PIEDA for four docking poses. (a) Pose 1 (b) Pose 2 (c) Pose 3 (d) Pose 4.

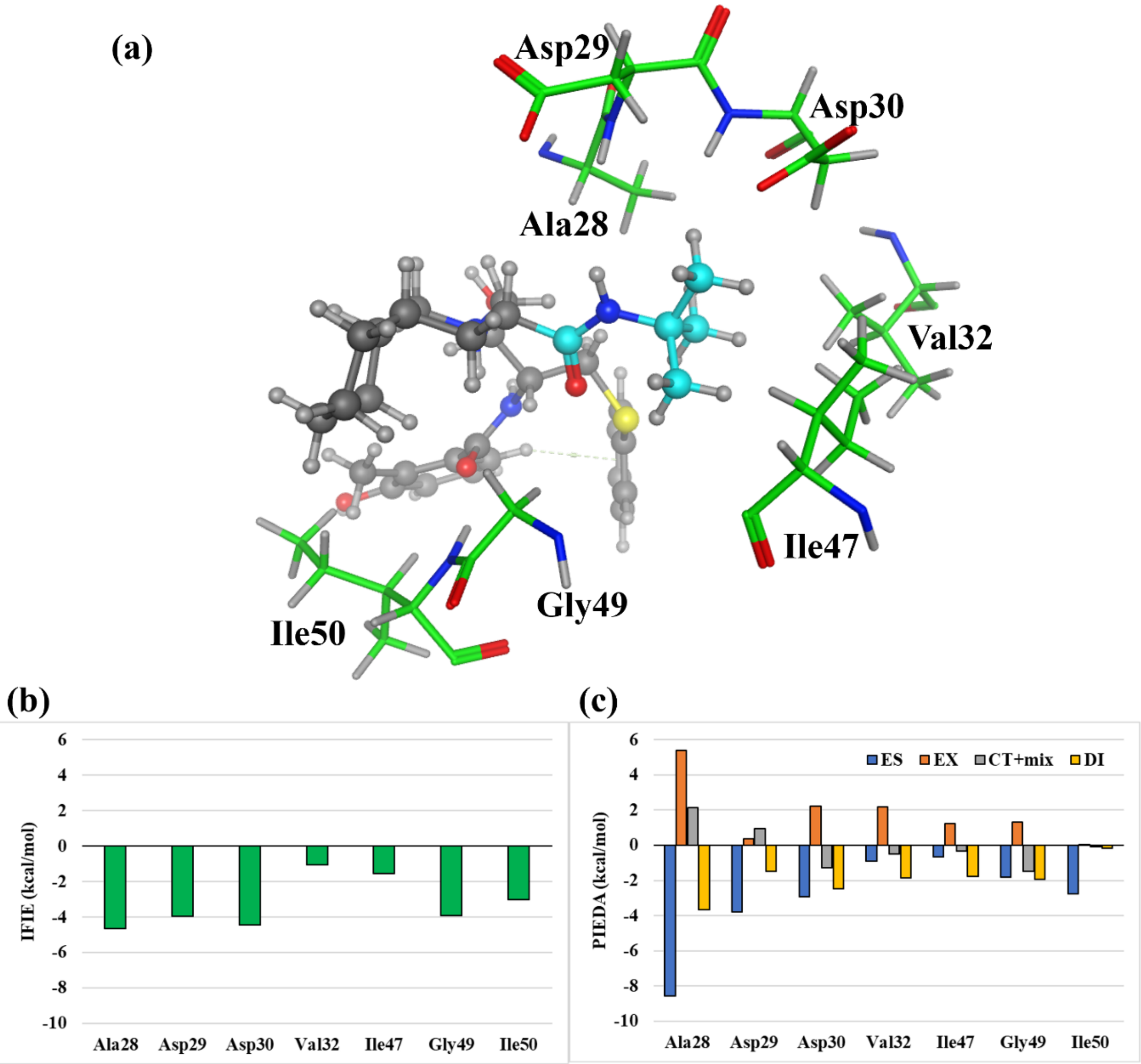

(d)

		ES	EX	CT + mix	DI	$\Delta E^{int(static)}$ (Total IFIE)
Energy between F4 of NLF and HIV-1 protease	F4	-15.9	17.4	-2.3	-19.8	-20.6
Energy between F4 of NLF and Mpro	Pose 1	-7.7 ± 7.5	12.5 ± 5.7	-4.2 ± 1.7	-14.3 ±2.9	-13.8 ± 6.6
	Pose 2	-6.8 ± 5.3	3.7 ± 3.6	-1.2 ± 1.4	-4.8 ± 3.4	-9.1 ± 5.7
	Pose 3	-18.0 ± 3.3	14.7 ± 4.5	-4.4 ± 1.1	-10.2 ± 1.8	-17.8 ± 2.8
	Pose 4	-10.3 ± 8.6	5.0 ± 4.6	-2.2 ± 1.5	-3.5 ± 1.7	-11.0 ± 7.7

Figure S5 The interaction involving the tert-butyl group of NLF. (a) Amino acid residues of HIV-1 protease interacting with the tert-butyl group of NLF (PDBID: 3el5). (b)(c) Interaction energy of each amino acid residue indicated by (a); (b) IFIE and (c) PIEDA energies. (d) Total IFIE and PIEDA of fragment containing tert-butyl groups (F4) (in kcal/mol).

Table S2 FMO calculation results for 15 structures of Mpro- Lopinavir complex obtained by docking (in kcal/mol), sorted by FMO score ($\Delta E^{int(static)}$). The six structures with the top FMO scores (excluding conformational overlap) were selected as Poses 1-6.

docking structure number	docking score	ES	EX	CT + mix	DI	$\Delta E^{int(static)}$ (Total IFIE)
No.2 (Pose 2)	-9.12	-91.0	93.0	-37.4	-93.5	-128.9
No.4 (Pose 4)	-8.88	-60.0	78.1	-40.7	-94.7	-117.3
No.5 (Pose 5)	-8.87	-62.6	64.8	-34.9	-84.3	-117.0
No.1 (Pose 1)	-9.14	-65.4	84.8	-36.9	-93.9	-111.4
No.9	-8.49	-65.6	79.4	-28.9	-90.6	-105.7
No.10 (Pose 6)	-8.45	-64.1	89.9	-34.7	-93.7	-102.7
No.3 (Pose 3)	-8.96	-65.4	83.4	-30.8	-89.0	-101.8
No.14	-8.43	-45.4	53.1	-27.8	-79.4	-99.5
No.8	-8.54	-54.7	68.1	-29.6	-81.4	-97.7
No.6	-8.63	-35.8	58.5	-30.9	-81.9	-90.1
No.13	-8.43	-52.1	72.1	-25.8	-83.9	-89.8
No.11	-8.44	-46.1	70.2	-28.2	-83.4	-87.5
No.12	-8.43	-36.5	72.6	-30.6	-88.7	-83.2
No.7	-8.63	-37.5	72.6	-26.2	-88.1	-79.2
No.15	-8.41	-36.7	72.1	-27.0	-83.0	-74.7

Table S3 Interaction energies of each docking pose in Lopinavir (in kcal/mol)

	Pose 1	Pose 2	Pose 3	Pose 4	Pose 5	Pose 6
ES	-53.7 ± 11.4	-76.8 ± 15.1	-37.1 ± 14.2	-45.4 ± 24.7	-46.0 ± 17.6	-43.5 ± 16.6
EX	55.7 ± 11.4	60.9 ± 16.0	38.5 ± 10.5	52.3 ± 18.6	51.0 ± 13.9	57.5 ± 15.6
CT + mix	-23.0 ± 3.5	-27.6 ± 4.8	-17.9 ± 3.8	-22.7 ± 6.4	-21.9 ± 4.3	-24.5 ± 6.7
DI	-56.4 ± 6.7	-56.1 ± 7.6	-47.4 ± 8.1	-57.4 ± 12.5	-52.6 ± 7.8	-70.3 ± 13.4
ΔE^{int}(Total IFIE)	-77.4 ± 11.1	-99.6 ± 12.0	-63.9 ± 15.1	-73.2 ± 25.7	-69.5 ± 15.6	-80.1 ± 23.1

Table S4 Energies (ΔE^{int}) between NFV and amino acid residues important for binding that are common to all binding poses and distances between their nearest neighboring atoms.

		Glu47	Asp48	Glu166	Asp187	Gln189
Pose 1	Distance (Å)	4.4 ± 1.7	6.7 ± 1.9	3.0 ± 0.9	3.0 ± 0.7	F3: 2.4 ± 0.2<br>F4: 2.8 ± 0.8
	ΔE^{int} (kcal/mol)	-31.1 ± 12.3	-20.6 ± 2.4	-10.7 ± 6.2	-26.4 ± 2.7	-14.7 ± 16.3
Pose 2	Distance (Å)	4.0 ± 1.0	6.4 ± 1.1	4.3 ± 1.8	2.6 ± 0.4	F1: 2.6 ± 0.7<br>F2: 2.4 ± 0.3
	ΔE^{int} (kcal/mol)	-39.6 ± 9.4	-26.0 ± 5.7	-26.1 ± 6.7.	-29.9 ± 3.2	-18.8 ± 10.5
Pose 3	Distance (Å)	4.3 ± 1.1	4.3 ± 1.3	1.9 ± 0.7	6.5 ± 0.8	F3: 1.9 ± 0.1<br>F4: 1.9 ± 0.1
	ΔE^{int} (kcal/mol)	-33.1. ± 8.0.	-39.4 ± 7.1	-49.6 ± 10.9	-34.4 ± 3.4	-45.2 ± 4.6
Pose 4	Distance (Å)	3.5 ± 0.3	5.2 ± 0.4	2.5 ± 0.2	3.0 ± 0.4	F1: 2.1 ± 0.3
	ΔE^{int} (kcal/mol)	-23.2 ± 3.4	-25.3 ± 1.6	-25.0 ± 3.4	-33.5 ± 1.8	-25.9 ± 6.6

Table S5. List of FMODB IDs of FMO calculation results for the docking and MD sampling structures.

Time step (ps)	FMODBID Pose1	FMODBID Pose2	FMODBID Pose3	FMODBID Pose4
docking structure	9MM52	L7759	3772L	M99KZ
50100	398KL	8544Y	V5JM1	4953N
50600	JL749	G6ZZ1	YZJ32	K9J33
51100	N9JZQ	1788Z	528MZ	QYZ1Y
51600	854GY	V5JJ1	4987N	R9658
52100	G6Z81	YZJJ2	K9MN3	Z6KYN
52600	1785Z	5284Z	QYJQY	6NK6Z
53100	V5JV1	4985N	R9VR8	2J92R
53600	YZJ92	K9MJ3	Z613N	72QGK
54100	528YZ	QYJZY	6N89Z	MV43Z
54600	4986N	R9V68	2J86R	94ZG2
55100	K9M83	Z61KN	725LK	LZYJ9
55600	QYJMY	6N8KZ	MVJGZ	39LQL
56100	R9VM8	2J89R	94KJ2	JLN39
56600	Z61LN	725QK	LZ989	N9K1Q
57100	6N87Z	MVJ4Z	398YL	85R2Y
57600	2J8RR	94KZ2	JL7J9	G6JN1
58100	725NK	LZ9Y9	N9J5Q	17M4Z
58600	MVJ8Z	398LL	8543Y	V59R1
59100	94K72	JL7N9	G6Z91	YZ4Y2
59600	LZ9G9	N9JKQ	178ZZ	5242Z
60100	398ZL	854RY	V5J71	4959N
60600	JL7Y9	G6ZJ1	YZJL2	K9J93
61100	N9J8Q	178MZ	528KZ	QYZYY
61600	8547Y	V5J91	4984N	R9698
62100	G6ZR1	YZJ42	K9M63	Z6K6N
62600	178YZ	528VZ	QYJ3Y	6NKNZ
63100	V5JN1	498VN	R9VJ8	2J9JR
63600	YZJ12	K9ML3	Z61QN	72Q2K
64100	5285Z	QYJGY	6N8QZ	MV4VZ
64600	498YN	R9VG8	2J81R	94Z42
65100	K9MV3	Z615N	725RK	LZYZ9
65600	QYJLY	6N8VZ	MVJ7Z	39L9L
66100	R9VL8	2J83R	94K62	JLNL9
66600	Z61ZN	725VK	LZ929	N9K9Q
67100	6N84Z	MVJ1Z	3985L	85R5Y
67600	2J8MR	94KV2	JL7Z9	G6J61
68100	725KK	LZ939	N9JMQ	17M7Z
68600	MVJLZ	398VL	854ZY	V5951
69100	94KR2	JL799	G6ZK1	YZ4Z2
69600	LZ9V9	N9JGQ	178GZ	524JZ
70100	398ML	854VY	V5J11	495JN
70600	JL7V9	G6Z21	YZJK2	K9JG3
71100	N9JLQ	178LZ	528LZ	QYZVY
71600	854QY	V5JG1	498MN	R9688
72100	G6ZV1	YZJG2	K9M13	Z6KVN
72600	1789Z	5286Z	QYJ9Y	6NKYZ
73100	V5JY1	498ZN	R9V38	2J9NR
73600	YZJR2	K9M53	Z61MN	72Q6K
74100	528GZ	QYJ6Y	6N8JZ	MV4NZ
74600	498GN	R9V28	2J8ZR	94Z82
75100	K9MQ3	Z618N	7258K	LZYN9
75600	QYJ5Y	6N82Z	MVJKZ	39LJL
76100	R9V48	2J8QR	94K52	JLNQ9
76600	Z619N	7257K	LZ959	N9KNQ
77100	6N85Z	MVJYZ	3982L	85R9Y
77600	2J8GR	94K92	JL789	G6J51

78100	7254K	LZ949	N9JRQ	17MNZ
78600	MVJZZ	398RL	8541Y	V59Q1
79100	94KQ2	JL7G9	G6ZM1	YZ4V2
79600	LZ9R9	N9JQQ	178QZ	524NZ
80100	398GL	854MY	V5J41	495LN
80600	JL7K9	G6ZG1	YZJ22	K9J23
81100	N9J6Q	178KZ	524ZZ	QYZNY
81600	854KY	V5JL1	495RN	R96Y8
82100	G6Z71	YZJ82	K9J73	Z6KNN
82600	178VZ	5281Z	QYZ7Y	6NKGZ
83100	V5JZ1	4981N	R96N8	2J9LR
83600	YZJ72	K9M43	Z6KRN	72QMK
84100	528QZ	QYJ2Y	6NKLZ	MV49Z
84600	498KN	R9V78	2J97R	94ZM2
85100	K9MZ3	Z614N	72Q3K	LZY79
85600	QYJ4Y	6N81Z	MV46Z	39L7L
86100	R9VK8	2J8VR	94ZN2	JLN29
86600	Z61GN	7251K	LZYM9	N9K2Q
87100	6N8RZ	MVJQZ	39L3L	85RNY
87600	2J85R	94K12	JLN69	G6JY1
88100	725ZK	LZ9K9	N9KVQ	17M6Z
88600	MVJ2Z	3981L	85R8Y	V59K1
89100	94K22	JL759	G6JL1	YZ4N2
89600	LZ969	N9JYQ	17M2Z	524RZ
90100	3984L	854YY	V5981	495QN
90600	JL7R9	G6Z31	YZ462	K9JK3
91100	N9J7Q	1781Z	5249Z	QYZKY
91600	8546Y	V5J31	495NN	R96Q8
92100	G6Z41	YZJ52	K9JR3	Z6K7N
92600	1783Z	5287Z	QYZ8Y	6NKZZ
93100	V5J61	4982N	R9618	2J94R
93600	YZJM2	K9MY3	Z6K2N	72QYK
94100	5288Z	QYJRY	6NK3Z	MV4RZ
94600	4988N	R9VZ8	2J9KR	94ZL2
95100	K9MM3	Z61JN	72QJK	LZYQ9
95600	QYJJY	6N8MZ	MV4MZ	39LKL
96100	R9VV8	2J8YR	94ZY2	JLN49
96600	Z611N	7259K	LZYL9	N9KZQ
97100	6N88Z	MVJ5Z	39LNL	85RGY
97600	2J88R	94K32	JLNM9	G6J81
98100	7255K	LZ919	N9K3Q	17M5Z
98600	MVJJZ	3986L	85RJY	V59V1
99100	94KK2	JL719	G6JQ1	YZ492
99600	LZ999	N9J4Q	17MJZ	524YZ
100100	MVJRZ	3988L	854LY	V5921
100600	94KL2	JL779	G6Z11	YZ4Q2
101100	LZ9Q9	N9JJQ	178RZ	5243Z
